# Comprehensive Proteomics Reveals Pachymic Acid Targeting RBMX via hnRNPC Degradation in Colorectal Cancer

**DOI:** 10.3390/cells15211992

**Published:** 2026-10-31

**Authors:** Weichao Ling, Meijing Song, Xinlin Li, Yu Li, Yuhao An, Zhidong Chen, Zhijun Ruan, Yang Zhou, Linlin Lu, Zigang Li, Rui Wang

**Affiliations:** 1 School of Pharmaceutical Sciences, State Key Laboratory of Traditional Chinese Medicine Syndrome, Chinese Medicine Guangdong Laboratory (Hengqin), Guangzhou University of Chinese Medicine, Guangzhou 510006, China; 2 Pingshan Translational Medicine Center, Shenzhen Bay Laboratory, Shenzhen 518118, China; 3 Shenzhen Bay Laboratory, Shenzhen 518132, China; 4 State Key Laboratory of Chemical Oncogenomics, School of Chemical Biology and Biotechnology, Peking University Shenzhen Graduate School, Shenzhen 518055, China; 5 Laboratory of Advanced Theranostic Materials and Technology, Ningbo Institute of Materials Technology and Engineering, Chinese Academy of Sciences, Ningbo 315300, China; 6 Ningbo Cixi Institute of Biomedical Engineering, Ningbo 315300, China

**Keywords:** pachymic acid, colorectal cancer, RBMX, LiP-MS, PL-MS

## Abstract

Pachymic acid (**PA**), a major bioactive component of Poria cocos, suppresses colorectal cancer (CRC) proliferation and invasion by disrupting glycolysis. However, its antitumor activity and underlying mechanisms in vitro and in vivo remain unclear. RNA-binding proteins (RBPs) are key post-transcriptional regulators in CRC, but their mechanisms of action and therapeutic potential remain poorly understood. Here, we employed limited proteolysis-mass spectrometry (LiP-MS), a target deconvolution approach that does not require prior chemical modification, to profile changes in protein conformation within complex proteomes upon **PA** treatment in cells. Our findings revealed that RBMX (RNA-binding motif protein X-linked) acts as a target of **PA** and undergoes concentration-dependent conformational changes, as supported by binding validation and functional assays performed in vitro and in vivo. Furthermore, proximity-labeling mass spectrometry (PL-MS) analysis revealed that RBMX interacts with heterogeneous nuclear ribonucleoprotein C (hnRNPC). Functional assays demonstrated that **PA** treatment induces RBMX-dependent degradation of hnRNPC, thereby suppressing CRC cell proliferation and tumor growth. In summary, this study identifies RBMX as a molecular target of PA through LiP-MS, providing new insights into the mechanisms underlying PA-mediated CRC suppression and highlighting the RBMX–hnRNPC axis as an important mediator of its antitumor effects.

## 1. Introduction

Colorectal cancer (CRC), a highly prevalent malignancy among gastrointestinal cancers, poses a major public health challenge worldwide [1]. Despite substantial advances in the treatment of CRC and improvements in long-term survival, the clinical management of CRC remains challenging. Many long-term survivors continue to experience persistent treatment-related complications that adversely affect bowel, urinary, sexual, and psychological function, highlighting that CRC management should extend beyond tumor control to address treatment-related burden and long-term quality of life [2]. Therefore, identifying novel therapeutic targets and developing therapeutic strategies with well-defined mechanisms of action are essential for improving treatment outcomes in CRC.

From the perspective of TCM pathogenesis, persistent dampness interacts with phlegm turbidity and heat toxins, culminating in mass formation that fosters tumor development [3,4,5]. Therefore, the TCM treatment of CRC prioritizes the “reinforce-Qi, expel-pathogen” (Fuzheng Quxie) strategy, which focuses not only on pathogen elimination or alleviation, but also on restoring Qi balance and invigorating the spleen, aiming to dispel dampness, clear heat and toxins, reduce phlegm and eliminate pathological metabolites [6,7,8]. *Poria cocos* (Fuling), a traditional medicinal herb known for strengthening the spleen, promoting diuresis, and resolving phlegm, is clinically used in TCM-based therapeutic regimens for CRC to alleviate syndromes characterized by spleen Qi deficiency, dampness, and phlegm obstruction [9].

Pachymic acid (**PA**), a major triterpenoid isolated from *Poria cocos*, is widely recognized as a key bioactive constituent contributing to the antitumor effects of *Poria cocos* against CRC [10]. Accumulating evidence demonstrates that **PA** is able to markedly inhibit CRC cell proliferation and invasion, reprogram tumor energy metabolism, and disrupt multiple oncogenic signaling cascades [11]. These pharmacological actions are consistent with the traditional TCM perspective that phlegm-turbidity accumulation promotes tumor growth and metastasis. Despite these observations, the specific protein targets of **PA** and the precise molecular mechanisms underlying its anti-CRC activity remain largely undefined.

Natural products typically exert therapeutic effects through the coordinated modulation of multiple targets and signaling pathways [12], a feature that is advantageous for treating tumors characterized by complex and heterogeneous pathogenic processes [13,14]. Mass spectrometry (MS) offers powerful approaches for protein- and peptide-level analysis, facilitating target identification and mechanism-of-action studies of natural products [15,16,17]. Limited proteolysis-mass spectrometry (LiP-MS) is a label-free, proteome-wide strategy that enables unbiased identification of direct molecular targets and potential binding regions by capturing ligand-induced conformational changes in target proteins under near-physiological conditions [18,19]. Consequently, LiP-MS has emerged as a powerful tool for target discovery in natural product studies [20].

In this study, we employed a proteome-wide LiP-MS approach to establish RBMX as a binding target of **PA** in CRC cells. **PA** treatment significantly induced structural changes in RBMX, whose endogenous protein levels were lower in several CRC cell lines than in normal cells. Mechanistically, **PA** binding enhanced RBMX stability, thereby increasing its protein abundance both in vivo and in vitro, which was accompanied by a reduction in hnRNPC protein levels. Using APEX2 proximity labeling coupled with MS, we identified hnRNPC as a binding partner of RBMX. Consistent with these findings, in vitro analyses further validated the physical and functional association between RBMX and hnRNPC, suggesting that **PA**-mediated stabilization of RBMX promotes hnRNPC degradation, with both the ubiquitin-proteasome system (UPS) and the autophagy-lysosome pathway (ALP) implicated in this process. Together, these findings reveal a novel molecular mechanism underlying the antitumor activity of **PA**, whereby **PA** stabilizes RBMX and promotes hnRNPC degradation to suppress the proliferation, migration, and invasion of CRC cells. The study provides mechanistic insights into the potential anti-CRC effects of **PA** and supports further investigation of its therapeutic relevance.

## 2. Materials and Methods

### 2.1. Reagents and Antibodies

Pachymic acid (**PA**, Supplementary Figure S1a, catalog no. CFN99590) was purchased from ChemFaces (Wuhan, China). HCQ, MG132, and Baf-A1 were purchased from MedChemExpress (Monmouth Junction, NJ, USA). Other chemical reagents were obtained from Sigma-Aldrich (St. Louis, MO, USA). The primary antibodies against RBMX (AB190352) and FLAG tag (AB205606) were purchased from Abcam (Cambridge, UK), while those targeting hnRNPC (68447-1-IG), β-actin (66009-1-IG), LC3I/II (14600-1-AP), SQSTM1/p62 (18420-1-AP), and HA tag (66006-2-IG) were obtained from Proteintech (Rosemont, IL, USA).

### 2.2. Cell Culture

RKO, HCT116, 293T, HCoEpiC, HepG2, MCF-7, A375, and HeLa cells were purchased from the American Type Culture Collection (ATCC, Manassas, VA, USA). CT26-luc cells were obtained from Guangzhou University of Chinese Medicine (Guangzhou, China). Cells were cultured in the appropriate culture media supplemented with 10% fetal bovine serum and maintained at 37 °C in a humidified atmosphere containing 5% CO_2_. All cell cultures were routinely monitored for contamination and maintained according to standard cell culture procedures.

### 2.3. Cell Activity Assay

Cell viability was assessed using the Cell Counting Kit-8 following the manufacturer’s protocol. Briefly, cells were seeded in 96-well plates at a density of 5 × 10^3^ cells/well and allowed to adhere for 24 h. Cells were then exposed to **PA** for 24 or 48 h. After treatment, 10 μL CCK-8 reagent was added to each well, followed by incubation at 37 °C for 2 h. Absorbance was measured at 450 nm using a microplate reader. Untreated cells served as the control (100% viability). Cell viability was calculated as a percentage relative to the control group and expressed as the mean ± SD of three independent experiments. Statistical analysis was performed using one-way ANOVA.

### 2.4. Cell Migration Assay

Cell migration was evaluated using Transwell chambers (8.0-μm pore size; Corning, REF353097) without Matrigel coating. Briefly, 1 × 10^4^ cells suspended in serum-free medium supplemented with **PA** were added to the upper chamber. The lower chambers were filled with medium containing 10% FBS as a chemoattractant. After incubation at 37 °C with 5% CO_2_ for 48 h, the non-migrated cells on the upper surface of the membrane were gently removed using a cotton swab. The migrated cells on the lower surface were fixed with 4% paraformaldehyde, stained with 0.1% crystal violet, and imaged under a microscope. The number of migrated cells was quantified by counting cells in three randomly selected fields per chamber.

### 2.5. Cell Invasion Assay

Cell invasion was evaluated using Matrigel-coated Transwell chambers. Briefly, 1 × 10^4^ cells suspended in serum-free medium supplemented with **PA** were added to the upper chamber. The lower chambers were filled with medium supplemented with 10% FBS serving as a chemoattractant. Following incubation at 37 °C with 5% CO_2_ for 48 h, cells remaining on the upper surface of the membrane were gently removed using a cotton swab. The invaded cells on the lower surface were fixed with 4% paraformaldehyde for 15 min, stained with 0.1% crystal violet for 20 min, and photographed under a microscope. The number of invaded cells was quantified by counting cells in three randomly selected fields per chamber.

### 2.6. Colony Formation Assay

To evaluate clonogenic ability, cells were seeded into 6-well plates at a density of 500 cells/well and cultured in complete medium containing **PA** for 10 days. The culture medium was replaced every 3 days. Upon the formation of macroscopic colonies, the plates were washed twice with PBS, fixed with 4% paraformaldehyde for 15 min, and stained with 0.1% crystal violet for 20 min at room temperature. After washing with distilled water and air-drying, colonies containing >50 cells were manually counted, and representative images were captured.

### 2.7. Western Blotting

Total cellular proteins were extracted and quantified using a BCA assay kit. Equal amounts of proteins were separated by SDS-PAGE and subsequently transferred onto PVDF membranes via electroblotting. The membranes were blocked with 5% nonfat milk and then incubated with primary antibodies at 4 °C overnight. After three washes with TBST (Tris-buffered saline with 0.1% Tween-20), the membranes were probed with HRP-conjugated secondary antibodies at room temperature for 1 h, followed by detection using enhanced chemiluminescence (ECL) reagent.

### 2.8. Purification of GST-T1

The T1 fragment (amino acids 1–95 of RBMX) was cloned into the pET28a-GST vector. The constructed plasmid was then transformed into BL21(DE3) competent *E. coli* cells, and protein expression was induced using IPTG. Following induction, bacterial cells were harvested and resuspended in PBS buffer containing 500 mM NaCl. Cell lysis was performed by sonication. The resulting lysate was purified through sequential binding, washing, and elution steps using an ÄKTA purification system to obtain the target fusion protein.

### 2.9. Immunofluorescence

After 24 h of **PA** treatment, HCT116 cells were fixed with 4% paraformaldehyde and permeabilized using 0.15% Triton X-100. The cells were then washed with PBS and blocked with 3% BSA in PBS for 30 min. Following blocking, the cells were incubated with a primary antibody (1:400 dilution) at 4 °C overnight. Subsequently, the cells were incubated with a fluorescent secondary antibody, followed by Hoechst staining for nuclear visualization. Images were finally acquired using a confocal microscope.

### 2.10. Generation of Stable Knockdown Cell Lines

To establish stable knockdown cell lines, specific shRNA sequences targeting the gene of interest were selected and synthesized from the Merck shRNA library. These sequences were then cloned into the pLKO.1 vector. Subsequently, the constructed plasmids were co-transfected with packaging plasmids into 293T cells to produce lentiviral particles. Viral supernatants were collected after 48 h and used to infect the target cells. Following infection, the cells were selected with puromycin to obtain stable cell lines with knockdown of the target gene.

### 2.11. Molecular Docking

Molecular docking analysis was performed using the AutoDock Vina (version 1.2.5) software. The three-dimensional structure of the target protein was retrieved from the PDB database. The structure was cleaned up in PyMOL (version 3.1.9) by removing all solvent molecules, bound ligands, and non-standard residues. Polar hydrogen atoms were added, and Gasteiger charges were assigned using AutoDock Tools (version 1.5.7). A docking grid was defined around the protein’s active site, with specific dimensions and center coordinates. Docking calculations were carried out using the default parameters. Upon completion, the optimal docking pose was selected based on the calculated binding affinity and the analysis of its binding mode. The protein-ligand interactions were subsequently visualized and analyzed using PyMOL.

### 2.12. Molecular Dynamics Simulation

The crystal structure of the target protein was prepared by removing the original ligand and water molecules. Protonation states of protein residues at physiological pH were assigned using the PROPKA and Reduce tools. The ligand geometry was optimized, and its force field parameters were generated using the Antechamber tool with the GAFF force field, while the AMBER ff14SB force field was applied to the protein. The protein-ligand complex was solvated in a cubic periodic box of TIP3P water molecules, maintaining a minimum distance of 10 Å between the protein surface and the box edges. Sodium and chloride ions were added to neutralize the system charge and achieve a physiological ionic concentration of 0.15 M. The system underwent energy minimization for 50,000 steps. A two-step equilibration protocol was followed. First, under the NVT ensemble, the system was gradually heated to 300 K over 100–200 ps while applying positional restraints (5 kcal·mol^−1^·Å^−2^) on the protein backbone. Subsequently, under the NPT ensemble (1 atm), the system was equilibrated for approximately 500 ps with a gradual release of restraints. Production simulations were then performed in the NPT ensemble at 300 K (regulated by the Langevin thermostat) and 1 atm (maintained using the Parrinello-Rahman barostat) with a 2-fs integration timestep. A cutoff of 10 Å was applied to short-range non-bonded interactions, and long-range electrostatic interactions were handled using the Particle Mesh Ewald method. Depending on the study objectives, production runs were typically conducted for 100 ns or longer, with trajectories saved every 10–100 ps for subsequent analysis. Trajectory analyses included calculating the root mean square deviation (RMSD) and root mean square fluctuation (RMSF) of the complex, quantifying the frequency of key hydrogen bonds and contacts, performing cluster analysis on ligand binding poses, and estimating binding free energies using the MM/PBSA method.

### 2.13. Sample Preparation of LiP-MS

RKO cells were treated with varying concentrations of PA (0, 10, and 40 μM) for 24 h, then the cells were harvested and lysed via sonication in lysis buffer to extract total proteins. The limited proteolysis samples were incubated with proteinase K at an enzyme-to-substrate ratio of 1:100 (*w*/*w*) for 5 min at 37 °C. The reaction was terminated by heating the samples at 99 °C for 5 min, followed by cooling on ice. The samples were then diluted with UB solution (0.1 M Tris-HCl, pH 8.0, containing 8 M urea) to a final volume of 200 μL. Dithiothreitol (DTT) was added to a final concentration of 10 mM, and the samples were incubated at 37 °C for 30 min to reduce disulfide bonds. Subsequently, iodoacetamide (IAM) was added to a final concentration of 55 mM, and the samples were incubated for 45 min at room temperature in the dark. The samples were transferred to 10 kDa FASP tubes, and 200 μL of 50 mM NH_4_HCO_3_ solution was added. The tubes were centrifuged at 14,000× *g* at 4 °C, after which the supernatant was replaced with fresh 50 mM NH_4_HCO_3_ solution. The concentrated samples were then transferred to new centrifuge tubes. Trypsin (Pierce, Thermo Fisher Scientific) was added at an enzyme (μg) to substrate protein (μg) ratio of 1:50 in 200 μL of 50 mM NH_4_HCO_3_ solution. The mixture was added to the FASP tubes and incubated overnight at 37 °C on a shaker. After digestion, peptide fragments were collected by centrifugation. Tryptic digestion was terminated by adding 1% formic acid (FA), followed by desalting using in-house-prepared 3M™ Empore™ C18 StageTips. The desalted peptides were dried by vacuum centrifugation and quantified at 280 nm using a NanoDrop 2000 spectrophotometer (Thermo Scientific).

### 2.14. LC-MS/MS (DIA) Analysis and Data Processing

Peptide samples were loaded onto a Thermo C18 analytical column (75 μm i.d. × 25 cm) driven by an Easy-nLC 1200 nano-liquid chromatography pump coupled online with an Orbitrap Exploris™ 480 mass spectrometer (Thermo Fisher Scientific, Waltham, MA, USA). Mobile phases A and B consisted of 0.1% FA in water and 0.1% FA in 80% acetonitrile (ACN), respectively. Chromatographic separation was achieved using a step-linear gradient: from 8% to 24% ACN over 103 min, followed by an increase in mobile phase B from 24% to 40% over 8 min. MS data were acquired in data-independent acquisition (DIA) mode. The key parameters for full MS scans (MS1) included a scan range of 350–1200 *m*/*z*, an Orbitrap resolution of 120,000, and a maximum injection time of 50 ms. For tandem MS scans (MS2), the default charge state was set to 3, and precursor ions were fragmented using a three-step collision energy of 25%, 30%, and 35%. All mass spectra were recorded in centroid mode.

MS raw data were acquired using Thermo Xcalibur 4.7 and processed via a library-free approach within FragPipe (v20.0) [21]. Briefly, a predicted spectral library was generated based on the “Human20230619.fasta” database using deep learning neural network algorithms. DIA data were searched using MSFragger-DIA, followed by spectral library refinement and peptide/protein quantification using DIA-NN, generating quantitative matrices at both the precursor and protein levels. The Match Between Runs (MBR) feature was enabled, and the false discovery rate (FDR) was strictly controlled below 1% at both the protein and precursor ion levels. Protein quantification was performed using the MaxLFQ algorithm implemented in the R package (v1.9.12). To rigorously control for missing values, only proteins fully identified across all three technical replicates in at least one experimental group were retained for further analysis. Differential protein abundance between groups was assessed using the limma package, and two-sided statistical tests were performed to calculate *p* values and fold changes (FC). *p* values were adjusted for multiple testing using the Benjamini–Hochberg (BH) method.

### 2.15. FASP Sample Preparation for LFQ Proteomics Across CRC Lines

The collected cells were resuspended in ice-cold RIPA lysis buffer supplemented with 1% (*v*/*v*) protease inhibitor cocktail and 1 mM PMSF, and incubated on ice for 10 min prior to sonication. Subsequently, the lysate was centrifuged at 12,000 rpm for 15 min at 4 °C to collect the supernatant containing total proteins. To precipitate the proteins, five volumes of ice-cold acetone/ethanol/acetic acid (*v*/*v*/*v* = 50/50/0.1) were added to the supernatant, and the mixture was incubated at −20 °C. The protein precipitate was harvested by centrifugation at 14,000× *g* for 30 min and then resuspended in 50 mM NH_4_HCO_3_ solution. Protein concentration was determined using the Bradford assay.

For protein digestion, the samples were reduced with 50 mM NH_4_HCO_3_ containing 8 M urea and DTT at 37 °C for 10 min, followed by alkylation with 30 mM iodoacetamide (IAM) for 30 min at room temperature in the dark. The samples were then diluted with 50 mM NH_4_HCO_3_ to a final urea concentration of 2 M. Trypsin was added at an enzyme-to-substrate ratio of 1:50 (*w*/*w*), and the mixture was incubated overnight at 37 °C. The digestion was terminated by adding FA to a final concentration of 1% (*v*/*v*). The resulting peptides were desalted and vacuum-dried for subsequent LC-MS/MS analysis.

### 2.16. Sample Preparation of APEX2-Based Proximity Labeling

HCT116 cells were transfected with the RBMX-APEX2 plasmid. After 48 h, the cells were treated with 0.5 mM BP (biotin phenol) for 30 min, followed by 1 min of incubation with H_2_O_2_. The reaction was quenched by washing three times with the quenching solution. Cells were harvested, resuspended in PBS containing PMSF, and sonicated on ice. Protein concentration was determined, and incubated with streptavidin-conjugated agarose beads at 4 °C overnight. Then, wash the beads with PBS with 0.1% TritonX-100, shake the beads for 5 min on a shaker and centrifuge each time; repeat five times. The labeled proteins on the beads were reduced by DTT, treated with IAM, and digested at 37 °C overnight by trypsin into peptides. The supernatant was collected by centrifugation (1000 *g*, 1 min) and combined with 2 × 50 μL water after washing beads, then acidified to a final concentration of 2% (*v*/*v*) FA and desalted for LC-MS/MS analysis.

### 2.17. LC-MS/MS (LFQ) Analysis and Data Processing

Peptide samples were loaded onto a Thermo C18 analytical column driven by an Easy-nLC 1200 nano-liquid chromatography system with an Orbitrap Exploris 480™ mass spectrometer for online analysis. Chromatographic separation was performed at a flow rate of 300 nL/min, utilizing a mobile phase system consisting of buffer A (0.1% FA in water) and buffer B (0.1% FA in 80% ACN). A linear gradient was applied to increase the ACN concentration from 8% to 40%. Data acquisition was executed in a data-dependent acquisition (DDA) Top N mode. MS1 scans were acquired at a resolution of 60,000 (at *m*/*z* 200) across a scan range of *m*/*z* 350–1800, with an automatic gain control (AGC) target set to 3 × 10^6^. Following each full scan, the top 20 most intense precursor ions were selected for MS/MS analysis through a 1.6 *m*/*z* isolation window. Precursor ions were fragmented via higher-energy collisional dissociation (HCD), and the resulting product ion spectra were recorded at a resolution of 15,000. Notably, single-charged ions and ions with unassigned charge states were dynamically excluded from MS/MS fragmentation.

Proteomic data were processed using MaxQuant (v2.4.9.0) against the UniProtKB reviewed human database (version 20230619). Mass tolerances for MS1 and MS2 were set to 10 ppm and 0.02 Da, respectively. Search parameters included trypsin digestion (up to 2 missed cleavages, a minimum length of 7 amino acids), carbamidomethylation of cysteine (fixed modification), and protein N-terminal acetylation, along with methionine oxidation (variable modifications). Both peptide and protein FDR were controlled within 1% using a target-decoy (reversed) database strategy. Additionally, the “Second peptides”, “Match between runs”, and “Dependent peptides” options were enabled, and LFQ was employed to determine abundance differences among the pull-down samples. Statistical significance of the differentials was evaluated using the limma R package, followed by Gene Ontology (GO) functional enrichment analysis based on the UniProt database.

### 2.18. An Orthotopic CRC Animal Model to Evaluate the Effects of PA

Male BALB/c mice were acclimated for 1 week, after which five mice were randomly selected and subcutaneously injected with 1 × 10^6^ CT26-luc cells. Following 7 days of additional housing, subcutaneous tumors were surgically excised, minced into 1 mm^3^ fragments, and preserved on ice for immediate orthotopic transplantation into additional BALB/c mice. For orthotopic implantation, mice were anesthetized with 1.5% isoflurane. A 1 cm abdominal incision was made to expose the peritoneum, and the cecum was exteriorized. Tumor fragments were sutured onto the outer serosal layer of the cecum using 7.0 surgical sutures. The cecum was carefully repositioned into the abdominal cavity, and the peritoneal incision was closed with 5.0 sutures in a continuous pattern from the inner to outer layer. Three days post-surgery, mice were randomly divided into four groups (n = 6): model group (vehicle control), OXA group (positive control), and **PA** groups (20, 40 mg/kg). Drugs were administered daily via oral gavage for 9 days, and body weight was monitored throughout the study. At the endpoint, tumors were excised, photographed, and weighed. Collected tumor tissues were divided into two aliquots: one aliquot was snap-frozen in liquid nitrogen and stored at −80 °C for subsequent molecular analyses, while the other was fixed in 4% paraformaldehyde at 4 °C for paraffin embedding and histological sectioning.

### 2.19. Statistical Analysis

All data are expressed as mean ± SEM. Each experiment was performed in at least three independent replicates. Statistical analyses were conducted using Prism Statistics software. Differences among multiple groups were assessed by one-way analysis of variance (ANOVA), followed by pairwise comparisons using Student’s *t*-test. A *p*-value < 0.05 was considered statistically significant.

## 3. Results

### 3.1. PA Exhibits Anti-Tumor Activity Both In Vitro and In Vivo

**Figure 1:**
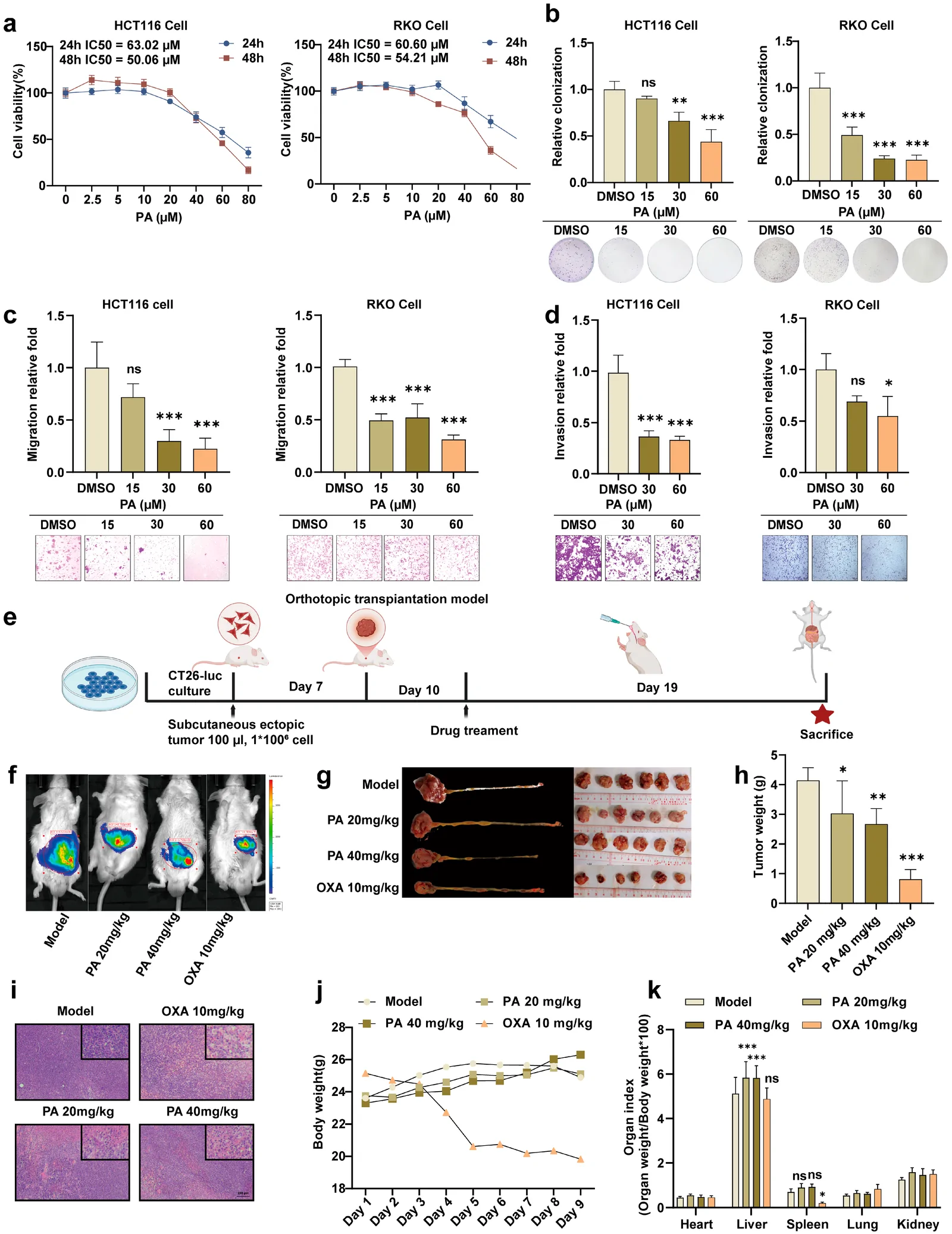
**PA** inhibits CRC progression in vitro and in vivo. (**a**) **PA** inhibits the viability of HCT116 and RKO cells in a dose-dependent manner. (**b**) **PA** suppresses colony formation in HCT116 and RKO cells. (**c**) **PA** significantly inhibits the migratory capacity of HCT116 and RKO cells. (**d**) **PA** markedly impairs the invasive ability of HCT116 and RKO cells. (**e**) Schematic illustration of the in vivo experimental design for evaluating the inhibitory effects of **PA** on CRC. (**f**) In vivo imaging demonstrates that **PA** effectively suppresses the progression of CRC. (**g**) **PA** significantly reduces tumor volume in an orthotopic CRC mouse model. (**h**) **PA** significantly decreases tumor weight in the animal model. (**i**) H&E staining reveals that **PA** treatment induces pronounced pathological alterations in tumors from the orthotopic CRC model. (**j**) Changes in body weight of mice during **PA** administration. (**k**) Alterations in organ indices following **PA** treatment. Statistical significance compared with the control group: ns, not significant; * *p* < 0.05; ** *p* < 0.01; *** *p* < 0.001. Note: IC_50_: half-maximal inhibitory concentration.

To assess the anti-tumor activity of **PA**, we first evaluated its cytotoxic effect on different human cancer cell lines. **PA** exerted minimal inhibitory effects on breast cancer, hepatocellular carcinoma, melanoma, and cervical cancer cells, whereas it displayed a growth-inhibitory activity against CRC cells (Figure 1a; Supplementary Figure S1c,d). In contrast to HCoEpiC cells, **PA** significantly decreased cell viability of HCT116 and RKO cells, with 24 h IC_50_ values of 63.02 μM and 60.60 μM, and 48 h IC_50_ values of 50.06 μM and 54.21 μM, respectively, indicating both the tumor selectivity and the toxicity profile (Figure 1a; Supplementary Figure S1b). Consistently, in colony formation assays, **PA** also markedly inhibited the clonogenic capacity of CRC cells (Figure 1b). Moreover, the transwell migration and invasion assays showed the impaired migratory and invasive abilities of CRC cells by **PA** (Figure 1c,d). Collectively, these results indicate that **PA** effectively suppresses malignant phenotypes of CRC cells in vitro.

In vivo, the orthotopic CRC mouse model was generated using CT26-luc cells. Three days after tumor implantation, mice were treated with **PA** (20 or 40 mg/kg) or oxaliplatin (OXA) (10 mg/kg) for nine consecutive days (Figure 1e). Both of them significantly suppressed tumor growth in vivo imaging (Figure 1f). Evidenced by decreased tumor volume and weight at necropsy, **PA** or OXA treatment further suppressed tumor growth in vivo, but **PA** exhibited a relatively weaker tumor suppression than OXA (Figure 1g,h). In addition, Hematoxylin and eosin (H&E) staining of tumor tissues displayed typical pathological alterations after **PA** or OXA treatment, including extensive necrosis, decreased cellular density, and nuclear fragmentation (Figure 1i). Notably, the body weight of **PA**-treated mice showed no change compared with control mice, whereas OXA caused a pronounced weight loss (Figure 1j). Organ index analysis further indicated that **PA** treatment mildly increased the liver index without concomitant weight loss, suggesting favorable overall tolerability. In contrast, OXA significantly reduced the kidney index and was associated with marked body weight reduction, reflecting higher systemic toxicity (Figure 1k). Altogether, these findings demonstrate that **PA** exerts anti-tumor effects against CRC both in vitro and in vivo, while being relatively well tolerated under the experimental conditions tested, and support further investigation of its therapeutic potential.

### 3.2. RBMX Is Critical for PA Pharmacological Efficacy

**Figure 2:**
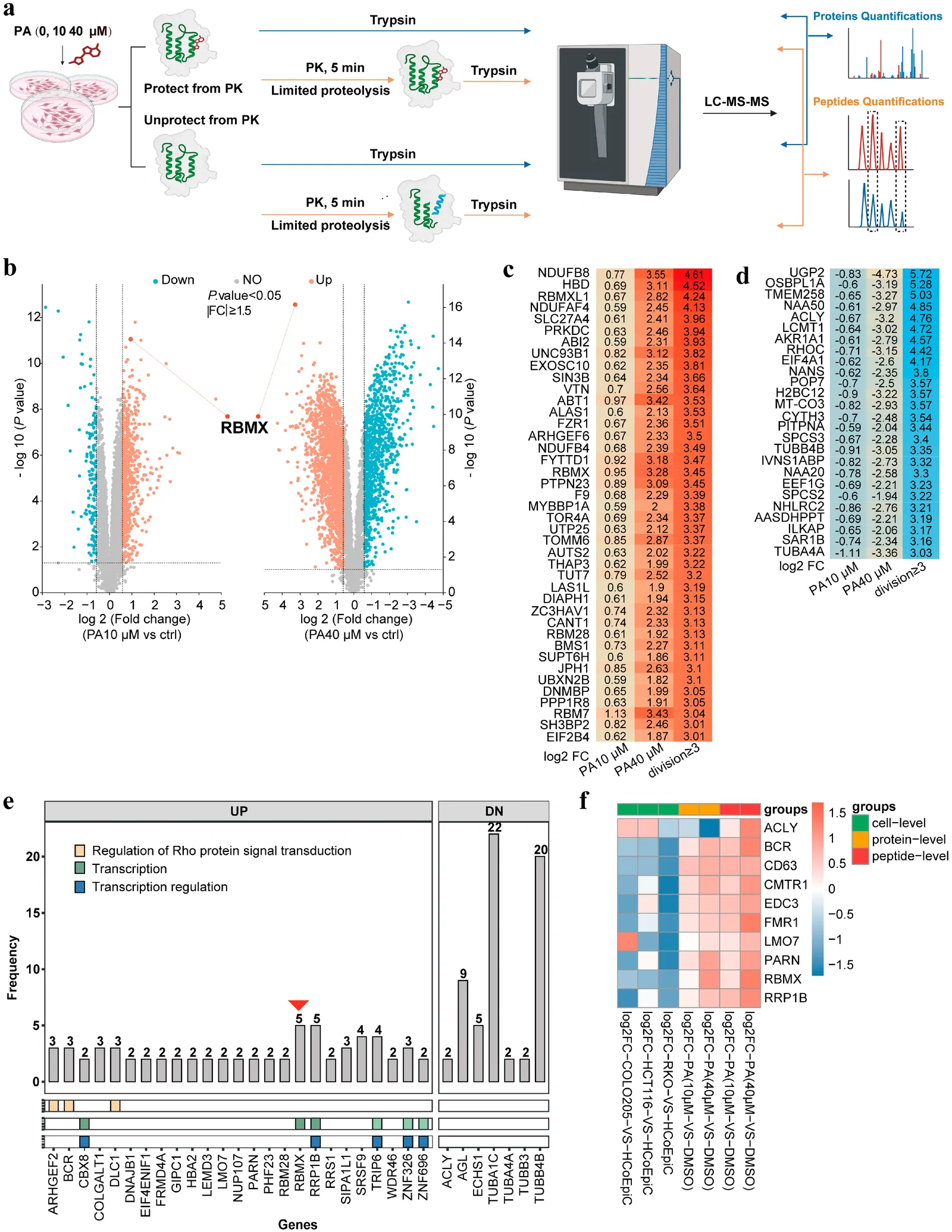
Identification of the PA binding target. (**a**) LiP-MS workflow used to identify **PA** binding targets. (**b**) Number of proteins exhibiting significant abundance changes following **PA** treatment (0, 10, and 40 μM). (**c**) The increase in protein abundance observed under the 40 μM and 10 μM PA treatment conditions. division = FC (40 μM PA/DMSO)/FC (10 μM PA/DMSO). (**d**) The decrease in protein abundance observed under the 40 μM and 10 μM PA treatment conditions. division = FC (40 μM PA/DMSO)/FC (10 μM PA/DMSO). (**e**) Proteins containing more than two identical differential peptides under **PA** dose-dependent treatment. (**f**) Label-free quantitative proteomics by FASP showing protein abundance changes across CRC cell lines and protein- and peptide-level alterations in RKO cells after **PA** treatment. Note: (**e**): the red triangle indicates RBMX, the selected candidate gene.

Next, to elucidate the molecular events under **PA** treatment, we employed LiP-MS without prior chemical modification to detect altered biological processes through probing structural changes directly on a proteome-wide scale. Herein, cells were treated with **PA** (10 or 40 μM) or vehicle control and underwent a brief double-protease digestion step by DIA quantification for the identification of structurally altered peptides and differentially abundant proteins (Figure 2a). For data filtering, differential analysis was performed using the limma package, with a fold change (FC) ≥ 1.5 and *p* ≤ 0.05 set as the initial thresholds. Under these criteria, **PA** induced dose-dependent proteomic changes, with 10 μM increasing 662 proteins and decreasing 144, and 40 μM increasing 1944 proteins while decreasing 919 relative to control. (Figure 2b). Intersection analysis of the two dose conditions revealed 585 proteins that were consistently upregulated and 94 proteins that were consistently downregulated across both concentrations (Supplementary Figure S1e). Given the importance of dose-dependent responses in LiP-MS target prioritization, as demonstrated by previous multi-point dose–response studies [22,23], we further evaluated the dose-dependent response to **PA** in our dataset. We introduced an additional ratio-based criterion, defined as division ≥ 3 (division = FC (40 μM **PA**/DMSO)/FC (10 μM **PA**/DMSO)), to prioritize structurally altered peptides and differentially abundant proteins showing a marked difference between the two **PA** concentrations. Using this criterion, **RBMX** exhibited a division value of **3.45** between the 10 and 40 μM **PA** treatment groups, supporting a pronounced dose-dependent change in RBMX protein abundance (Figure 2c,d, Supplementary Table S1). In **PA**-induced structural alterations (Supplementary Figure S1f), proteins with more than two peptides showing dose-dependent changes were further selected (Figure 2e). Notably, five changed peptides of RBMX were found under two conditions, suggesting that **PA** binding induces local conformational changes or alters protease-accessible cleavage sites within RBMX (Supplementary Table S2). GO and KEGG enrichment analyses revealed that the differentially affected proteins were predominantly enriched in pathways related to transcriptional regulation and RNA splicing (Supplementary Figure S1g). Similarly, FASP-based label-free quantitative proteomics across CRC lines revealed that RBMX, BCR, and CD63 were significantly downregulated in common to control HCoEpiC cells (Supplementary Table S3), whereas **PA** treatment consistently increased their protein abundance and the levels of differential peptides (Figure 2f). Combined with pathway enrichment, dose-dependent responses, and biological relevance, we prioritized the RNA-binding protein RBMX as a key candidate for following validation.

### 3.3. PA Binds to the RRM Domain of RBMX

**Figure 3:**
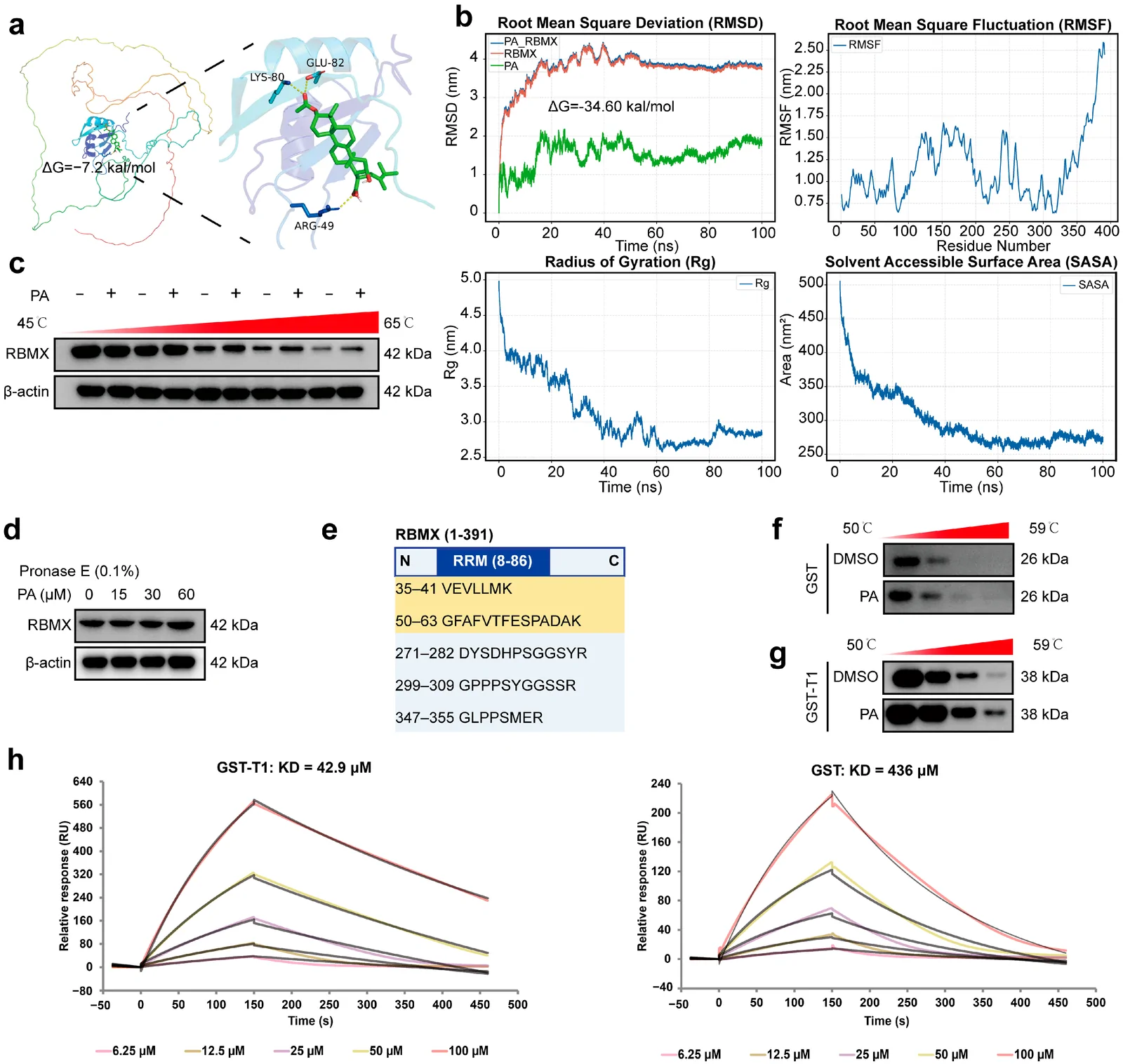
PA binds to the RRM domain of RBMX. (**a**) Molecular docking simulation of the binding affinity between **PA** and RBMX. (**b**) Molecular dynamics simulation of the **PA**-RBMX complex. (**c**) CETSA validation of the interaction between **PA** and RBMX. (**d**) DARTS validation of the interaction between **PA** and RBMX. (**e**) Schematic representation of the RBMX structure and the specific peptides derived from RBMX identified in LiP-MS analysis. (**f**) CETSA analysis of the interaction between **PA** and GST (negative control). (**g**) CETSA analysis of the interaction between **PA** and the T1 fragment. (**h**) SPR analysis validating the direct binding of **PA** to T1, with GST serving as a control. Note: (**e**): yellow-highlighted peptides indicate peptides showing statistically significant differences following both 10 and 40 μM PA treatments, whereas blue-highlighted peptides indicate peptides showing statistically significant differences only following 40 μM PA treatment.

Alongside the binding between **PA** and RBMX, we employed molecular docking to evaluate their potential binding. Based on the structural information of RBMX (PDB: AF_AFP38159F1), the molecular docking calculations indicated a binding affinity of −7.2 kcal/mol for **PA** to RBMX, suggesting a strong binding capability (Figure 3a). Building upon this, we further conducted molecular dynamics (MD) simulations to assess the stability of the binding complex. The RMSD analysis revealed that the RBMX-**PA** complex stabilized after approximately 15~20 ns, with **PA** remaining within the predicted binding pocket without significant dissociation. The calculated binding free energy (ΔG = −34.60 kcal/mol) further supported the stability of the complex. RMSF analysis indicated that most amino acid segments of RBMX (residues 1–300) exhibited fluctuations within a range of 0.5~1.5 nm, suggesting that **PA** binding primarily influences the conformational stability of this region. Concurrently, Rg of the complex decreased from an initial value of approximately 5.0 nm and stabilized between 2.7~3.0 nm, indicating that RBMX adopted a more compact and stable conformation upon **PA** binding. The SASA remained stable within the range of 260~280 nm^2^, also supporting the overall structural stability of the complex (Figure 3b). Collectively, these computational results suggest a stable binding interaction between **PA** and RBMX. To further experimentally confirm their interaction, we performed Cellular Thermal Shift Assay (CETSA) and Drug Affinity Responsive Target Stability (DARTS) assays. The CETSA results demonstrated that **PA** enhanced the thermal stability of RBMX in a dose-dependent manner (Figure 3c, Supplementary Figure S1h). DARTS analysis also showed that **PA** treatment increased the resistance of RBMX to Pronase E digestion (Figure 3d**,** Supplementary Figure S1i). Both findings are characteristic of enhanced conformational stability upon ligand binding, supporting the interaction between **PA** and RBMX. To further delineate the binding region, we integrated the differential peptides of RBMX identified in the LiP-MS data, which were primarily clustered within the regions of 35~63 aa and 271~355 aa (Figure 3e). Analyzing the structural features of RBMX, we noted that its N-terminal region (1~95 aa) contains a highly conserved RRM. Both molecular docking and MD simulations suggested that the **PA** binding site was likely located within this domain. Therefore, we expressed and purified the T1 fragment (RBMX_T1) encompassing this region (Supplementary Figure S1j). CETSA performed on the purified T1 protein showed that **PA** significantly enhanced its thermal stability (Figure 3f,g). Furthermore, SPR measurements indicated a binding affinity (KD) of 42.9 µM for **PA** to the T1 fragment, compared to a much weaker affinity of 436 µM for the negative GST protein, further demonstrating the specific binding of **PA** to the RRM domain of RBMX (Figure 3h). Taken together, both computational simulations and multiple experimental lines of evidence support that **PA** binds to RBMX, primarily targeting its N-terminal RRM domain.

### 3.4. PA Inhibits CRC Progression via RBMX

**Figure 4:**
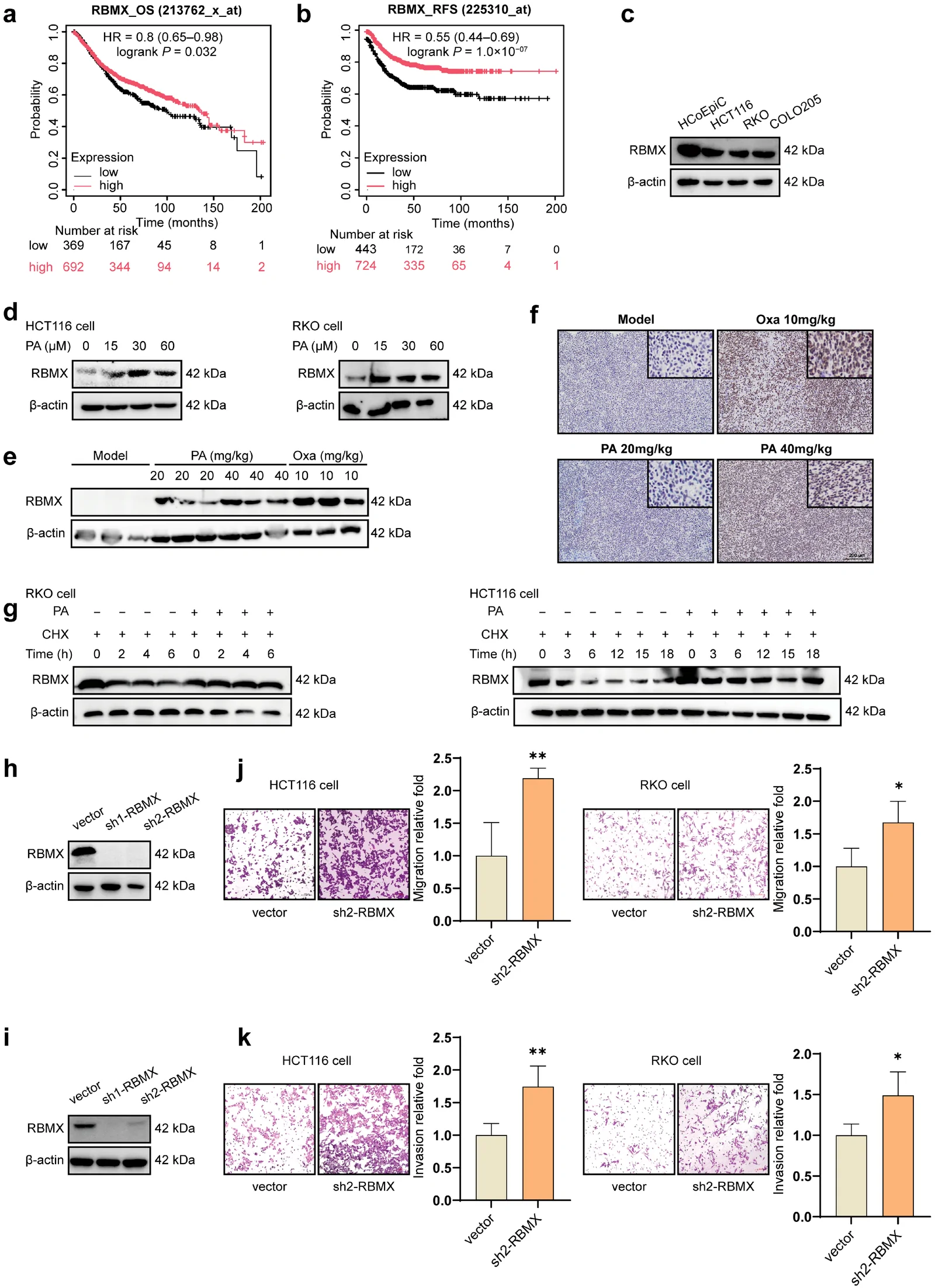
RBMX is a tumor suppressor gene in CRC. (**a**) Kaplan–Meier Plotter analysis of the correlation between RBMX expression levels and OS in patients with CRC. (**b**) Kaplan–Meier Plotter analysis of the correlation between RBMX expression levels and RFS in patients with CRC. (**c**) Western blot analysis of RBMX protein expression in CRC cell lines. (**d**) RBMX protein levels in HCT116 and RKO cells following **PA** treatment for 48 h. (**e**) Western blot analysis of RBMX expression in tumor tissues from animals. (**f**) IHC analysis of RBMX expression in tumor tissues from treated animals. (**g**) CHX chase assays evaluating the effect of **PA** treatment on RBMX protein stability in HCT116 and RKO cells. (**h**) Validation of stable RBMX knockdown in HCT116 cells. (**i**) Validation of stable RBMX knockdown in RKO cells. (**j**) Enhanced migratory capacity of HCT116 and RKO cells following RBMX knockdown. (**k**) Enhanced invasive capacity of HCT116 and RKO cells following RBMX knockdown. Statistical significance compared with the control group: * *p* < 0.05; ** *p* < 0.01.

To elucidate the relationship between RBMX and CRC under **PA** treatment, we first investigated the Kaplan–Meier survival analysis using the KM-plotter database, revealing that high RBMX expression was significantly associated with improved overall survival (OS) and relapse-free survival (RFS) in patients with CRC (Figure 4a,b). Consistently, RBMX protein expression was markedly reduced across three commonly used CRC cell lines (Figure 4c). Further analysis of the Human Protein Atlas (HPA) database revealed reduced RBMX protein expression in a subset of colorectal cancer tissue samples, further supporting the possibility that RBMX downregulation may be associated with CRC progression (Supplementary Figure S1k). An in vitro study demonstrated that dose-dependent **PA** treatments significantly increased intracellular RBMX protein levels, although quantitative PCR analysis showed no appreciable change in RBMX mRNA expression, indicating that **PA** regulates RBMX primarily at the post-translational level (Figure 4d; Supplementary Figure S1l,m). Furthermore, analysis of tumor tissues from **PA**-treated animals revealed that RBMX protein expression was elevated in both **PA** and OXA treatments, as determined by Western blotting and immunohistochemistry (IHC), with OXA exerting a more pronounced effect (Figure 4e,f). These observations are consistent with the anti-tumor efficacy of OXA and **PA** observed in vivo. Cycloheximide (CHX) chase assays further demonstrated that **PA** treatment prolonged the half-life of RBMX protein, suggesting that the improved protein stability of RBMX by **PA** increased its protein level (Figure 4g). To explore the influences of RBMX on malignant phenotypes of CRC cells, we established stable RBMX knockdown in HCT116 and RKO cell lines (Figure 4h,i). Functional assays revealed that RBMX depletion significantly enhanced the migratory and invasive capacities of CRC cells, revealing a negative regulatory role for RBMX in cell migration and invasion-associated phenotypes (Figure 4j,k). Collectively, these findings indicated that **PA** increases intracellular RBMX protein levels by improving its stability, thereby contributing to the suppression of malignant phenotypes in CRC.

### 3.5. Interaction Between RBMX and hnRNPC

**Figure 5:**
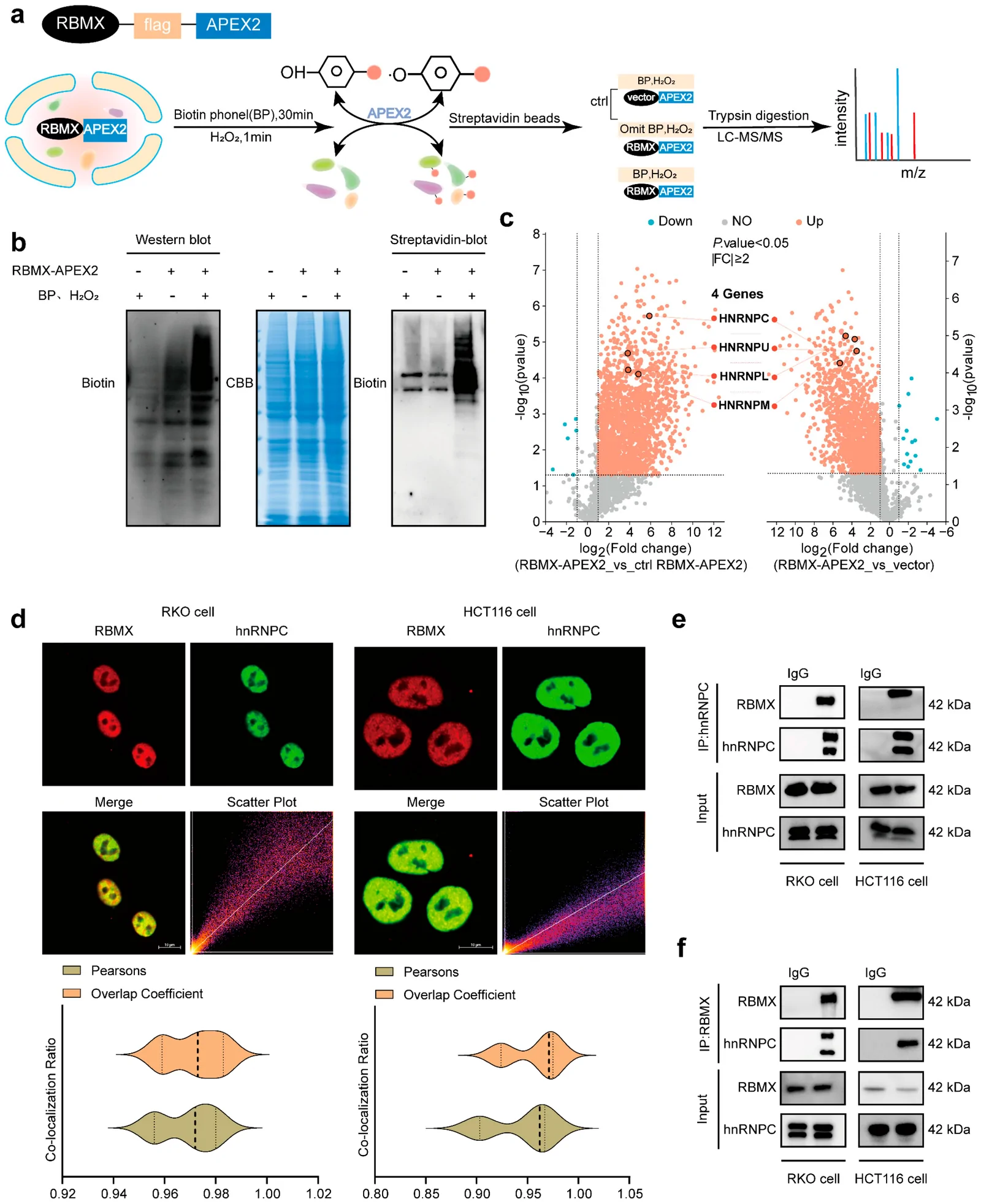
Interaction between RBMX and hnRNPC. (**a**) Schematic of the RBMX-APEX2 proximity labeling assay. (**b**) Western blot analyses verifying the functionality of the RBMX-APEX2 system. (**c**) Differentially enriched proteins identified by PL-MS analysis. (**d**) Immunofluorescence assay showing the co-localization of RBMX and hnRNPC. (**e**) Immunoprecipitation of RBMX in RKO and HCT116 cells to examine the interaction between RBMX and hnRNPC. (**f**) Immunoprecipitation of hnRNPC in RKO and HCT116 cells to assess its interaction with RBMX. Note: (**a**): the red circles represent the biotin moieties of biotin-phenol (BP), whereas the other colored shapes represent different proteins.

To systematically map cell signaling events and protein–protein interaction (PPI) networks of RBMX in living cells, we designed the RBMX-APEX2 for RBMX-proximity labeling of its interactome. HCT116 cells were transfected with the RBMX-APEX2 plasmid for 48 h, incubated with biotin-phenol for 30 min, and subsequently stimulated with hydrogen peroxide (H_2_O_2_) for 1 min. In each sample condition, biotinylated proteins were then specifically captured by streptavidin beads followed by label-free quantitative mass spectrometry analysis (Figure 5a). We first assessed the promiscuous labeling activity of RBMX-APEX2 by Western blot, showing H_2_O_2_-dependent biotinylation (Figure 5b). Three biological replicates showed good consistency without significant outliers, indicating the data were suitable for subsequent analysis (Supplementary Figure S2a–c). Upon H_2_O_2_ activation, substantially more proteins were identified in the experimental group compared with the two negative controls lacking either the targeting vector or H_2_O_2_ (Figure 5c, Supplementary Table S4). A PPI network was constructed based on the STRING database with an initial confidence threshold of 0.4 (Supplementary Figure S2d). When the confidence threshold was raised to 0.9 to filter for high-confidence interactions, cytoscape-based topological analysis using betweenness centrality identified hnRNPC and hnRNPM among the highest-ranked candidates (Supplementary Figure S2e). Notably, hnRNPC exhibited a higher fold change than hnRNPM in the PL-MS analysis, which provided additional support for prioritizing hnRNPC for subsequent experimental validation (Supplementary Table S4). Patchway enrichment analysis further indicated that these candidate proteins were primarily involved in RNA binding and splicing-related pathways (Supplementary Figure S2f). Consequently, we focused subsequent functional validation on selected hnRNPC. Immunofluorescence microscopy showed a high degree of nuclear co-localization between RBMX and hnRNPC (Figure 5d). Although the Co-IP assay also presents physical and associative contacts between RBMX and hnRNPC (Figure 5e,f), they did not regulate their protein levels (Supplementary Figure S2g,h). Therefore, the specific regulatory mechanism of **PA** on CRC progression through this RBMX-hnRNPC axis remains to be further elucidated.

### 3.6. HnRNPC Promotes the Progression of CRC

**Figure 6:**
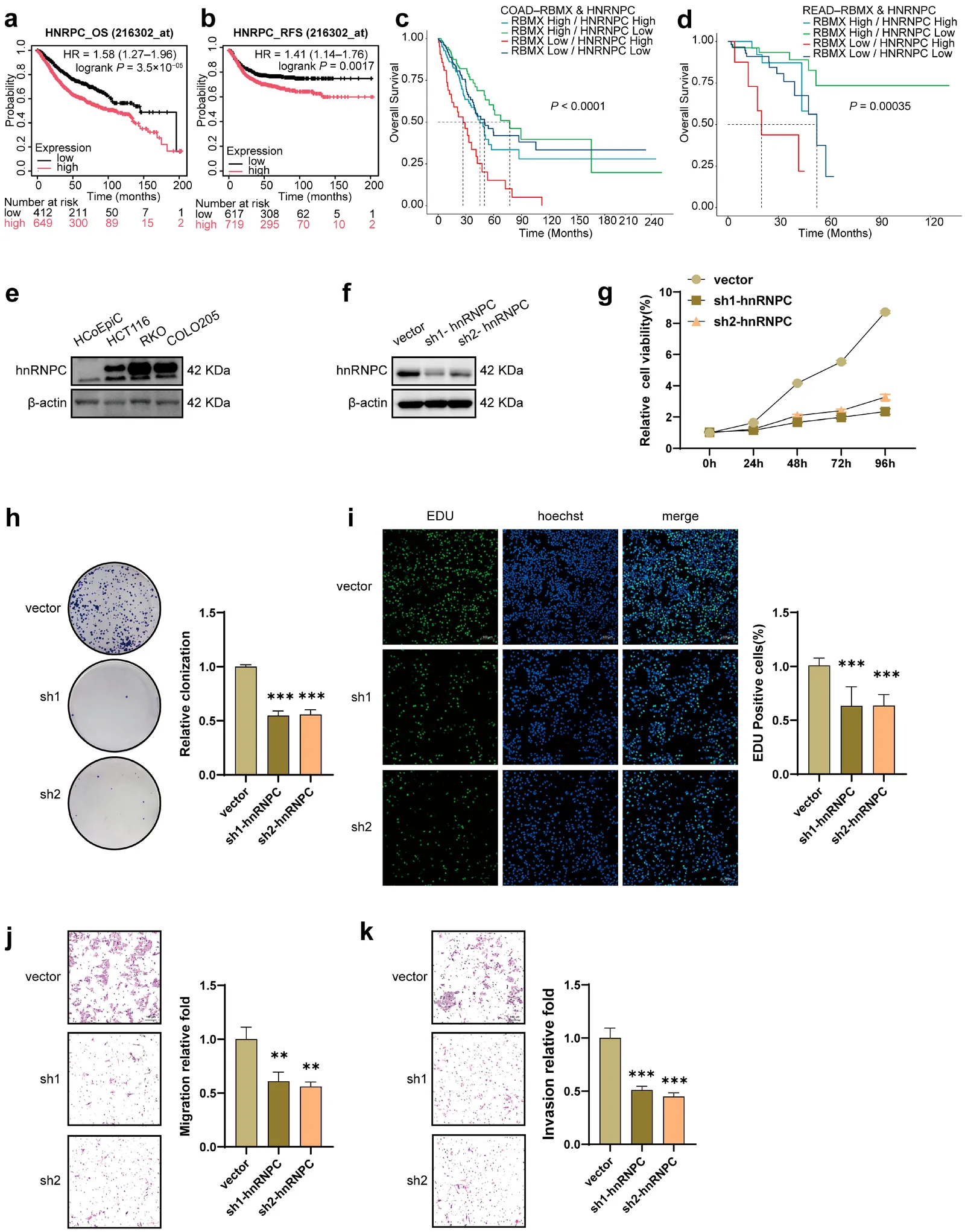
hnRNPC promotes the progression of CRC. (**a**) KM-plotter database analysis of the correlation between hnRNPC expression levels and OS in CRC patients. (**b**) KM-plotter database analysis of the correlation between hnRNPC expression levels and RFS in CRC patients. (**c**) TCGA database analysis of the correlation between the expression levels of RBMX and hnRNPC genes and overall survival in colon cancer patients. (**d**) TCGA database analysis of the correlation between the expression levels of RBMX and hnRNPC genes and overall survival in rectal cancer patients. (**e**) Western blot analysis of hnRNPC protein levels in a panel of CRC cell lines. (**f**) Validation of stable hnRNPC-knockdown in HCT116 cells. (**g**) Cell viability assay demonstrating the effect of hnRNPC knockdown on HCT116 cells. (**h**) Colony formation assay evaluating the effect of hnRNPC knockdown on the clonogenic ability of HCT116 cells. (**i**) EDU incorporation assay evaluating the effect of hnRNPC knockdown on the proliferative capacity of HCT116 cells. (**j**) Migration assay demonstrating the effect of hnRNPC knockdown on the migratory capacity of HCT116 cells. (**k**) Invasion assay demonstrating the effect of hnRNPC knockdown on the invasive capacity of HCT116 cells. Statistical significance compared with the control group: ** *p* < 0.01; *** *p* < 0.001.

Given the physical interaction between RBMX and hnRNPC, we wondered whether hnRNPC itself plays a pivotal role in CRC. KM-plotter analysis indicated that high expression of hnRNPC was associated with worse OS and RFS in CRC patients (Figure 6a,b). Further survival analysis of the TCGA (The Cancer Genome Atlas) identified a “high-RBMX/low-hnRNPC” expression signature correlated with the most favorable survival outcome in cancer. (Figure 6c,d). Across commonly used CRC cell lines, hnRNPC expression level was prominently increased (Figure 6e). To investigate the potential biological role of hnRNPC in tumor cell behavior, we successfully constructed stable hnRNPC-knockdown HCT116 cell lines (Figure 6f) followed by functional assays. Compared to the negative control vector, hnRNPC knockdown remarkably decreased cell viability and proliferation, and reduced cell migration and invasion capabilities (Figure 6g,k). These results support the notion that hnRNPC functions to promote proliferation and invasion in CRC cells, suggesting it acts as a pro-tumorigenic gene.

### 3.7. PA Suppresses CRC by Inducing hnRNPC Degradation.

**Figure 7:**
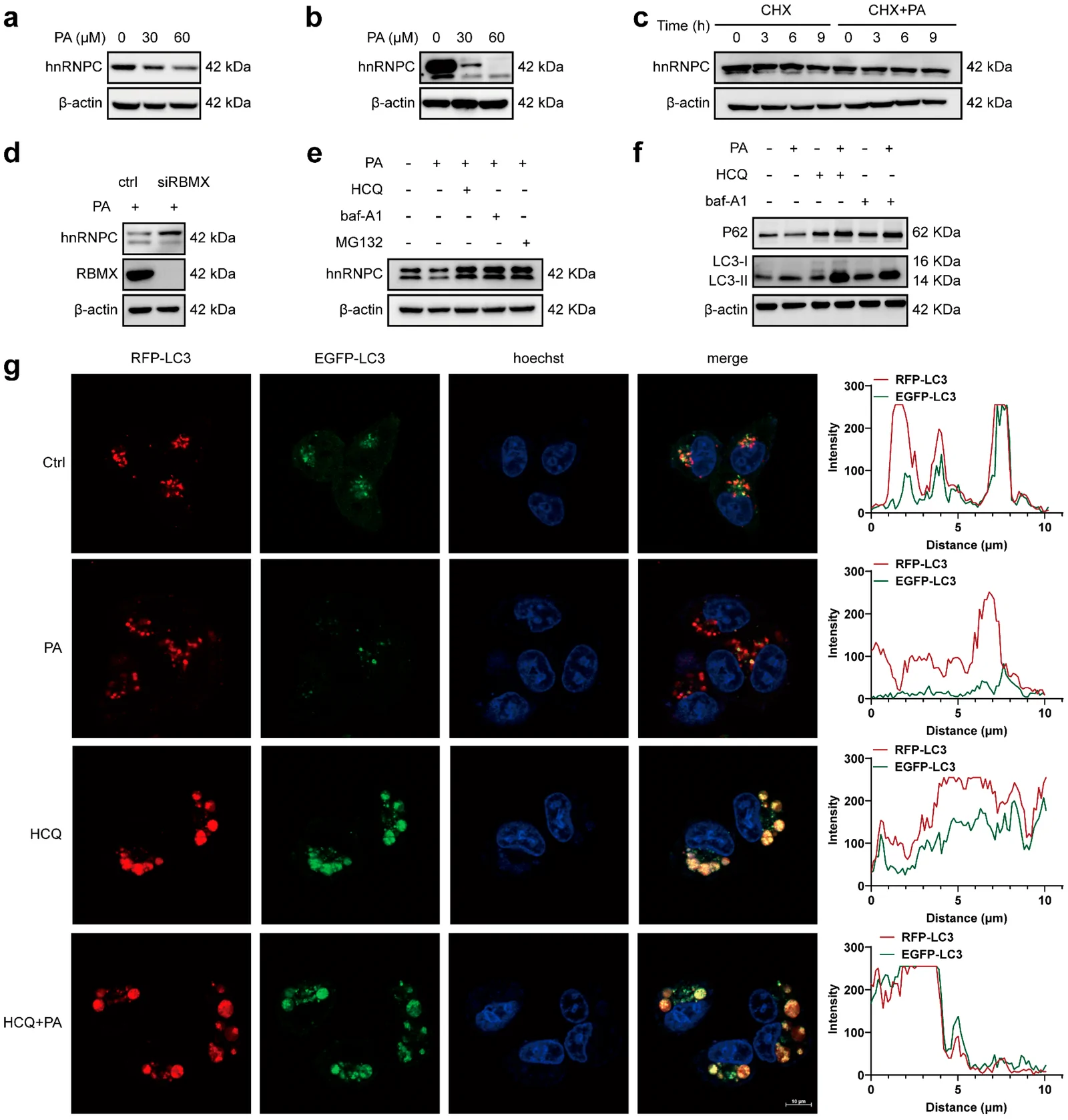
PA degrades hnRNPC in HCT116 cells via both the ubiquitin-proteasome and autophagy pathways. (**a**) hnRNPC protein levels after 24 h of **PA** treatment. (**b**) hnRNPC protein levels after 48 h of **PA** treatment. (**c**) CHX chase assay evaluating the effect of **PA** on hnRNPC protein stability. (**d**) Effect of **PA** on hnRNPC degradation following RBMX knockdown. (**e**) Effects of proteasome and autophagy pathway inhibitors on **PA**-induced hnRNPC degradation. (**f**) Impact of **PA** on autopha gy markers in cells. (**g**) Assessment of **PA**-induced autophagy using the RFP-EGFP-LC3 tandem reporter system.

Finally, to assess whether **PA** inhibits CRC through the RBMX-hnRNPC axis, we first examined the effect of **PA** on hnRNPC in HCT116 cells and observed a **PA** dose-dependent reduction in hnRNPC protein levels (Figure 7a for 24 h, 7b for 48 h; Supplementary Figure S3a), consistent with immunofluorescence imaging of a decreased hnRNPC signal intensity upon **PA** treatment (Supplementary Figure S3b). CHX chase assays showed that **PA** reduced the half-life of hnRNPC protein within 3 h in HCT116 cells (Figure 7c). In contrast, RBMX knockdown reversed the **PA**-induced reduction in hnRNPC protein levels (Figure 7d), whereas qPCR analysis showed no significant changes in hnRNPC mRNA levels (Supplementary Figure S3c,d). Moreover, CETSA and DARTS assays did not support an interaction between **PA** and hnRNPC (Supplementary Figure S3e,f). To further define the functional contribution of hnRNPC to the antitumor activity of **PA**, we performed hnRNPC overexpression rescue experiments. Overexpression of hnRNPC partially reversed the **PA**-mediated inhibition of CRC cell proliferation, indicating that hnRNPC is a key downstream target of the antitumor effects of **PA** (Supplementary Figure S3i–k). These findings demonstrate that **PA** could contribute to the regulation of hnRNPC primarily at a post-translational level. Not surprisingly, we observed an overall increase in cellular ubiquitination with **PA** treatment (Supplementary Figure S4a). In stable HCT116/hnRNPC-mCherry-HA cells, HA-IP detected significantly increased ubiquitination of hnRNPC upon **PA** treatment, alongside elevated RBMX abundance (Supplementary Figure S4b). Treatment with HCQ orBaf-A1, as well as the proteasome inhibitor MG132, attenuated **PA**-induced hnRNPC degradation (Figure 7e). These results suggest that PA-induced hnRNPC degradation is mediated through both the UPS and ALP. Consistent with these findings, **PA** treatment decreased p62 levels while increasing LC3-II levels, both of which are commonly used markers of autophagy (Supplementary Figure S4c). The reduction in p62 levels was reversed by co-treatment with HCQ or Baf-A1, indicating that **PA** enhances autophagic flux (Figure 7f). In stable cells expressing the tandem RFP-EGFP-LC3 reporter, **PA** treatment caused quenching of the GFP signal and an increase in red (RFP-only) puncta, while HCQ or HCQ + **PA** treatment significantly increased yellow puncta (GFP/RFP colocalization). These results further support that **PA** activates autophagy and promotes autophagosome-lysosome fusion and degradation (Figure 7g). Finally, in the EGFP-LC3 stable cell line, **PA** treatment increased the colocalization of EGFP-LC3 with lysosomal markers, corroborating that **PA** induces autophagy-mediated degradation (Supplementary Figure S4d). Therefore, our findings suggest that both the UPS and ALP contribute to PA-induced hnRNPC degradation.

**Figure 8:**
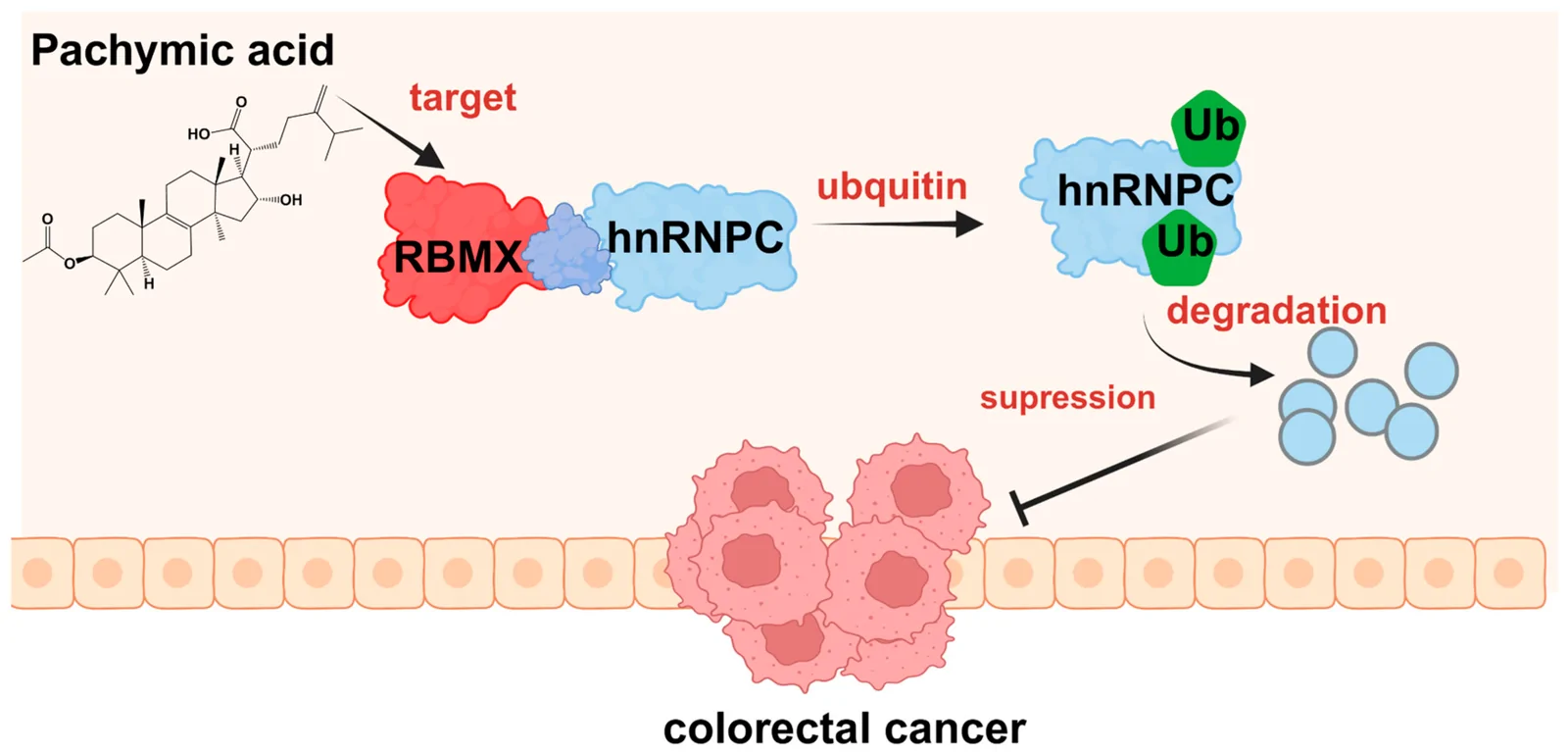
Mechanism Diagram. **PA** binds and stabilizes RBMX, while RBMX interacts with hnRNPC. Furthermore, **PA** promotes the degradation of hnRNPC through RBMX-mediated ubiquitin-proteasome and lysosome-autophagy pathways, thereby inhibiting the progression of CRC. Note: the blue circles indicate hnRNPC undergoing degradation.

In summary, our findings demonstrate that PA exerts anti-CRC effects in both in vitro and in vivo models. These effects were associated with increased RBMX protein levels and decreased hnRNPC protein levels. Although RBMX and hnRNPC showed an inverse relationship at the protein level, our results further revealed a physical and functional association between these two proteins. As a bioactive small molecule, PA showed relatively weak binding to RBMX but promoted RBMX protein stabilization, which was associated with promoted hnRNPC degradation through RBMX regulation and contributed to the suppression of CRC cell proliferation (Figure 8). Collectively, our study provides new mechanistic insights into the PA-RBMX-hnRNPC axis and identifies this pathway as a potential mechanism underlying the anti-CRC activity of PA, warranting further investigation.

## 4. Discussion

Previous studies have reported the antitumor effects of PA in various cancer cells. However, its role and underlying mechanisms in CRC remain poorly elucidated. In this study, we demonstrate that **PA** suppresses CRC progression both in vitro and in vivo and identify RBMX as a potential molecular target of **PA**. Mechanistically, **PA** promotes the degradation of hnRNPC, which is associated with the UPS and ALP. These findings reveal a previously unrecognized **PA**-RBMX-hnRNPC regulatory axis in CRC.

RBMX and hnRNPC are members of the RBP family, which plays important roles in post-transcriptional regulation and cancer progression. Dysregulation of RBPs has been implicated in tumorigenesis, metastasis, drug resistance, and metabolic reprogramming [24,25]. Our integrated analysis of global proteomic and LiP-MS data revealed that RBMX is downregulated in CRC cells, whereas **PA** treatment partially restored its protein abundance. Importantly, higher RBMX expression was associated with improved survival in CRC patients. Although RBMX has been reported to exert context-dependent functions across cancer types, including regulation of chromatin states, alternative splicing, transcription, and drug resistance [26,27], its functional role in CRC remains incompletely defined. These findings prompted us to investigate the RBMX protein interactome.

Using PL-MS, we identified several RBPs, including hnRNPC, hnRNPL, hnRNPM, and hnRNPU, as prominent components of the RBMX interactome. The enrichment of proteins involved in mRNA splicing and spliceosome function was consistent with the coordinated regulatory functions of RBPs. Among these candidates, hnRNPC was of particular interest because it was highly expressed in CRC and associated with unfavorable patient survival. Notably, combined survival analysis showed that patients with high RBMX and low hnRNPC expression had significantly longer overall survival. **PA** treatment reduced hnRNPC protein abundance without substantially affecting its mRNA level, suggesting that **PA** regulates hnRNPC primarily at the post-translational level. This finding is particularly relevant given the established oncogenic functions of hnRNPC in CRC, including its involvement in EMT, metastasis, metabolic reprogramming, and acquired drug resistance [28,29]. Thus, **PA**-mediated depletion of hnRNPC may represent an important mechanism underlying its antitumor activity.

Recent advances in targeted protein degradation have highlighted the therapeutic potential of eliminating oncogenic RBPs. PROTACs and molecular glues targeting RBPs such as LIN28 have demonstrated that selective protein degradation can suppress cancer-associated phenotypes [30]. However, the large size of PROTACs often results in poor solubility and limited cell permeability [31,32]. Moreover, the discovery of molecular glues remains largely serendipitous [33]. These challenges restrict their broader application. Here, we demonstrate that the natural product **PA** promotes hnRNPC degradation, an effect that is likely mediated by its interaction with RBMX. This novel mechanism, in which a drug binds one RBP to induce degradation of another, not only offers new insights for developing RBP-targeting therapeutics but also underscores the complexity of RBP regulatory networks.

Mechanistically, our data indicate that **PA**-induced hnRNPC degradation involves both the UPS and ALP. The involvement of multiple degradation pathways is consistent with the pleiotropic biological activities frequently observed for natural products, but it also underscores the complexity of their pharmacological mechanisms. Previous studies have shown that PA can suppress tumor-cell proliferation through cell-cycle regulation, particularly G1/S arrest, as well as metabolic and apoptotic mechanisms, including inhibition of aerobic glycolysis via the PPARγ/ENO1 pathway in colon cancer [10,11,34,35,36]. However, whether specific cell-cycle checkpoints, particularly the G1/S transition, are directly regulated downstream of the PA-RBMX-hnRNPC axis remains unclear and warrants further investigation.

The present study has several limitations. First, **PA** exhibited moderate antiproliferative activity against CRC cells, with IC_50_ values in the range of approximately 50–63 μM, and showed relatively weak binding affinity for RBMX in vitro. However, ligand-target affinity alone does not fully determine in vivo pharmacological efficacy, which is also influenced by drug exposure, tissue distribution, free drug concentrations at the target site, duration of exposure, and target occupancy [37,38]. Thus, the relatively weak **PA**-RBMX affinity observed in vitro should not be interpreted in isolation. Nevertheless, whether pharmacologically relevant concentrations of free **PA** can be achieved in tumor tissues remains unknown. Future pharmacokinetic and pharmacodynamic studies should therefore determine plasma and tumor **PA** exposure, free **PA** concentrations, and in vivo RBMX target engagement to establish a quantitative relationship between **PA** exposure, RBMX modulation, and antitumor efficacy. Second, although the PA-RBMX interaction was supported by multiple complementary approaches, including LiP-MS, SPR, CETSA, and DARTS, the lack of competition experiments with excess unlabelled PA means that the specificity of this target engagement remains to be further validated. Third, although pharmacological inhibition experiments support the involvement of the ALP in **PA**-induced hnRNPC degradation, genetic validation using core autophagy-related genes, such as ATG5 or ATG7, is currently lacking. Future studies employing genetic perturbation of these pathways will be required to further define the contribution of ALP to **PA**-induced hnRNPC degradation and to distinguish its role from proteasome-mediated degradation. Fourth, although we supplemented our analysis with publicly available HPA IHC data, the clinical relevance of the RBMX-hnRNPC axis requires further validation in independent human CRC specimens. CRC treatment outcomes are influenced by tumor-intrinsic alterations as well as the tumor microenvironment and immune architecture, and therapeutic resistance remains a major clinical challenge [39]. Although hnRNPC has been implicated in acquired therapeutic resistance in CRC [29], whether pharmacological modulation of the RBMX-hnRNPC axis by **PA** can enhance the efficacy of standard CRC therapies, such as 5-fluorouracil or oxaliplatin, or delay the development of acquired resistance remains to be determined. Future studies evaluating **PA** in combination with standard chemotherapy, particularly in treatment-resistant CRC models, will be important to determine whether **PA** has potential as a chemosensitizing agent or modulator of therapeutic resistance.

## 5. Conclusions

In this study, by integrating global proteomics with label-free LiP-MS, we identified RBMX as a potential protein target of PA. PA treatment partially restored RBMX protein abundance in CRC cells. Furthermore, RBMX-APEX2-based PL-MS identified hnRNPC, hnRNPL, hnRNPM, and hnRNPU as RBPs closely associated with the RBMX interactome. hnRNPC was highly expressed in CRC and was negatively associated with patient survival. Extensive in vitro and in vivo analyses demonstrated that PA reduced hnRNPC protein levels without significantly affecting its mRNA expression, suggesting that PA promotes hnRNPC degradation at the post-translational level. Moreover, pharmacological inhibition of either the proteasome or ALP attenuated PA-induced hnRNPC degradation, suggesting that both the UPS and ALP are involved in this process. Collectively, these findings support a model in which PA binding to RBMX promotes RBMX stabilization and is associated with promoted hnRNPC degradation, thereby contributing to the suppression of CRC progression.

## Data Availability

The mechanism diagram was created with BioRender.com. We would like to thank Servicebio Company for providing the H&E and IHC service. The mass spectrometry proteomics data have been deposited to the ProteomeXchange Consortium (http://proteomecentral.proteomexchange.org) via the iProX partner repository [40,41] with the dataset identifier PXD078463 (URL:02 May 2027). The data that support the findings of this study are available.

## Supplementary Materials

The following supporting information can be downloaded at: https://www.mdpi.com/article/10.3390/cells15211992/s1, Figure S1: The inhibitory effects of **PA** on multiple cancer cells and PA binding to RBMX; Figure S2: Quantification of RBMX in the PL-MS assay; Figure S3: HnRNPC is a key downstream target through which PA exerts its anti-colorectal cancer effects; Figure S4: In HCT116 cells, **PA** treatment enhances autophagic flux.; Table S1: Proteins FC(Lip-MS); Table S2: Peptides FC (LiP-MS); Table S3: DEPs_limma_CRC cell line; Table S4: RBMX-APEX2_PL-MS.
