# Supplementary material for: Comprehensive Proteomics Reveals Pachymic Acid Targeting RBMX via hnRNPC Degradation in Colorectal Cancer
Source: Cells. 2026 Oct 31;15(21):1992. doi: 10.3390/cells15211992 (PMC13761667; doi:10.3390/cells15211992)
Supplement: Supplementary file 1 [file cells-15-01992-s001.zip › Supporting information.pdf]

Supplementary Information

Comprehensive Proteomics Reveals Pachymic Acid Targeting RBMX via hnRNPC Degradation in Colorectal Cancer

Weichao Ling [a, b, c],Meijing Song [a], Xinlin Li [a, b, c], Yu Li [a], Yuhao An [b, c], Zhidong Chen [b, c, d], Zhijun Ruan [b], Yang Zhou [e], Linlin Lu [a], Zigang Li [b, c, d] *, Rui Wang [b, c] *

[a] School of Pharmaceutical Sciences, State Key Laboratory of Traditional Chinese Medicine Syndrome, Chinese Medicine Guangdong Laboratory (Hengqin), Guangzhou University of Chinese Medicine, Guangzhou, 510006, China.

[b] Pingshan Translational Medicine Center, of Shenzhen Bay Laboratory, Guangdong Shenzhen 518118, China.

[c] Shenzhen Bay Laboratory, Shenzhen, 518132, China.

[d] State Key Laboratory of Chemical Oncogenomics, School of Chemical Biology and Biotechnology, Peking University Shenzhen Graduate School, Shenzhen 518055, China.

[e] Laboratory of Advanced Theranostic Materials and Technology, Ningbo Institute of Materials Technology and Engineering, Chinese Academy of Sciences, Ningbo, 315300, China; Ningbo Cixi Institute of Biomedical Engineering, Ningbo, 315300, China.

*Corresponding authors.

E-mail addresses: lizg@pkusz.edu.cn (Zigang Li), wangrui@szbl.ac.cn (Rui Wang)

Table of contents

Supplementary Figures: (PDF)

Supplementary Figure S1. The inhibitory effects of **PA** on multiple cancer cells and PA binding to RBMX.

Supplementary Figure S2. Quantification of RBMX in the PL-MS assay.

Supplementary Figure S3. HnRNPC is a key downstream target through which **PA** exerts its anti-colorectal cancer effects**.**

Supplementary Figure S4. In HCT116 cells, **PA** treatment enhances autophagic flux.

Supplementary Tables: (XLSX)

Supplementary Table S1-1. Differentially abundant proteins after **PA** treatment（PA10 μM vs DMSO）

Supplementary Table S1-2. Differentially abundant proteins after **PA** treatment（PA40 μM vs DMSO）

Supplementary Table S2-1. Dose-dependent quantification of peptides upon treatment with **PA** (10 μM vs DMSO)

Supplementary Table S2-2. Dose-dependent quatification of peptides upon treatment with **PA** (40 μM vs DMSO)

Supplementary Table S2-3. LiP-MS results showing peptides of RBMX exhibiting dose-dependent changes upon PA treatment

Supplementary Table S3. Proteome-wide label-free quantification for CRC and HCoEpiC cells

Supplementary Table S4. Endogenous proteins identified by RBMX-APEX2 proximity labeling

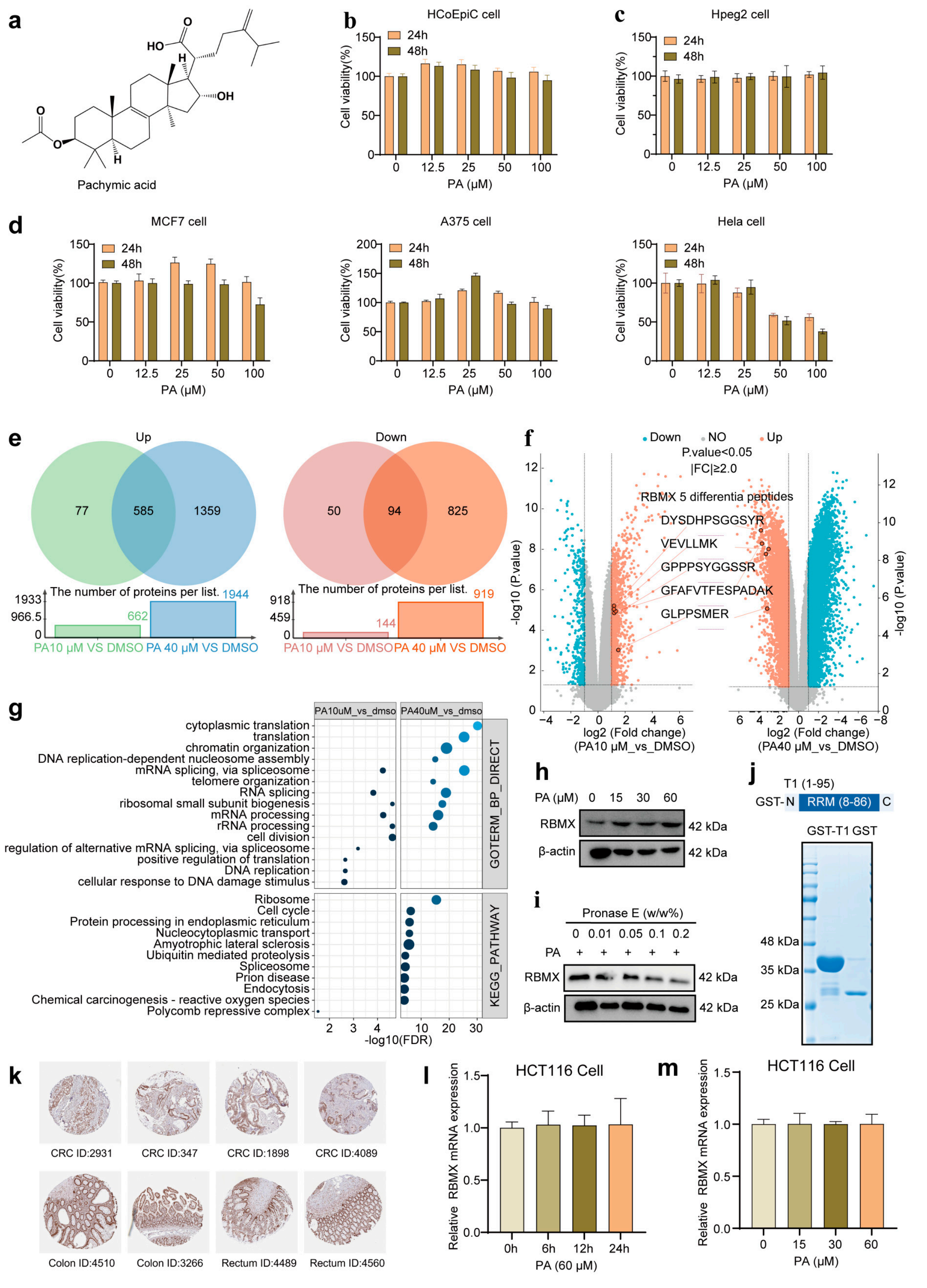

Supplementary Figure S1. The inhibitory effects of **PA** on multiple cancer cells and PA binding to RBMX. **a** Chemical structure of Pachymic acid (**PA**) monomer. **b** **PA** has no significant effect on the viability of HCoEpiC cells. **c** Effects of **PA** on cell viability in hepatocellular carcinoma as determined by the CCK-8 assay. **d** Effects of **PA** on cell viability in breast cancer, melanoma, and cervical cancer cells as determined by the CCK-8 assay. **e** Proteins showing significant abundance changes in RKO cells

following **PA** treatment (0, 10, and 40 μM). **f** Significantly altered peptides identified in RKO cells following **PA** treatment. **g** Gene enrichment analysis of proteins showing significant changes in the LiP-MS dataset. **h** CETSA assessing the effect of increasing concentrations of **PA** on RBMX thermal stability. **i** DARTS assay evaluating the effect of increasing concentrations of Pronase E on RBMX protease susceptibility. **j** Expression and purification of recombinant RBMX-T1 and GST proteins. **k** Analysis of the HPA database revealed reduced RBMX protein expression in human colorectal cancer tissue samples. **l** Effects of **PA** treatment at different time points on RBMX mRNA levels in HCT116 cells. **m** Effects of increasing concentrations of **PA** on RBMX mRNA levels in HCT116 cells after 24 h of treatment.

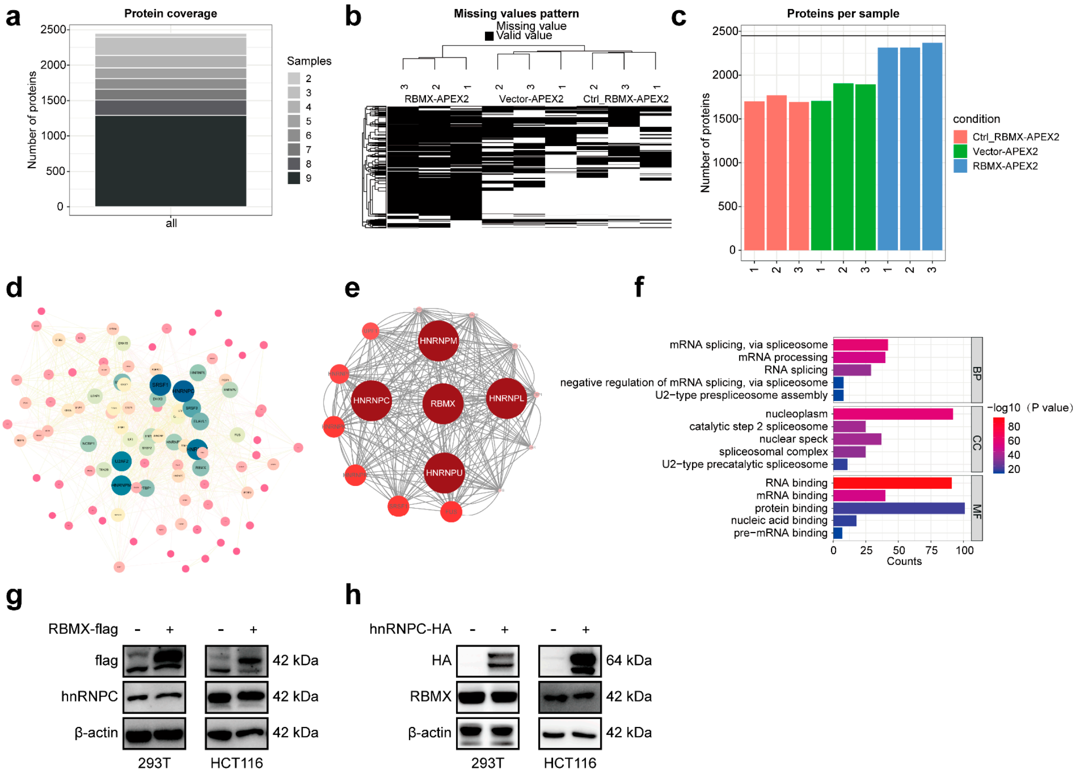

Supplementary Figure S2. Quantification of RBMX in the PL-MS assay. **a** Protein sequence coverage analysis of the PL-MS data. **b** Number of proteins identified in each sample in the PL-MS data. **c** Number of identified proteins per sample in the PL-MS data. **d** PPI network constructed from differentially enriched proteins identified by PL-MS using the STRING database (confidence score, 0.4). **e** PPI network constructed from differentially enriched proteins identified by PL-MS using the STRING database (confidence score, 0.9). **f** GO enrichment analysis of proteins identified by PL-MS. **g** Overexpression of RBMX-flag in 293T and HCT116 cells and its effect on hnRNPC

protein levels. **h** Overexpression of hnRNPC-mcherry-HA in 293T and HCT116 cells and its effect on RBMX protein levels.

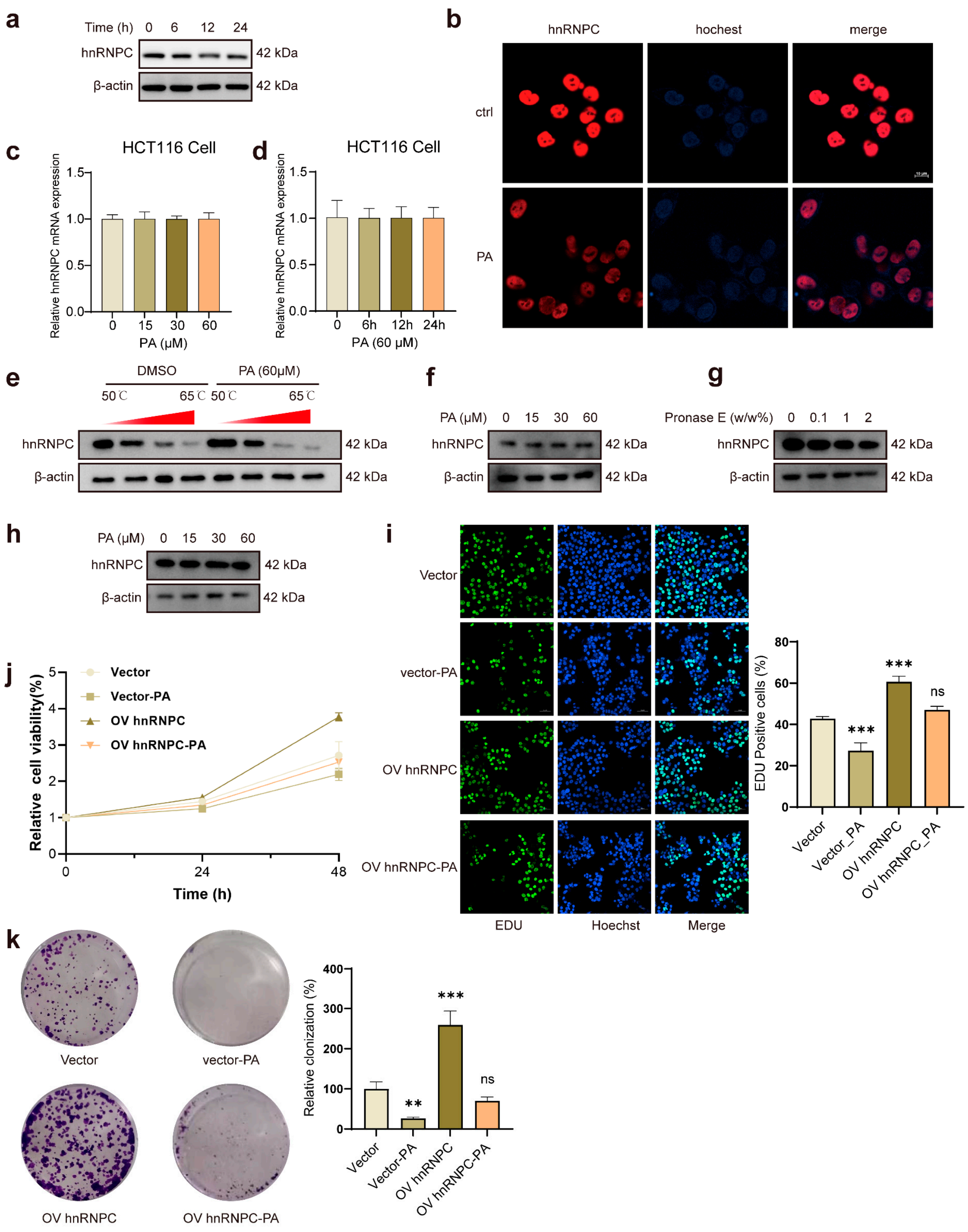

Supplementary Figure S3. HnRNPC is a key downstream target through which **PA** exerts its anti-colorectal cancer effects**. a PA** treatment significantly decreased hnRNPC protein abundance in HCT116 cells. **b** Immunofluorescence analysis of hnRNPC protein levels in HCT116 cells treated with 60 μM **PA** for 24 h. **c** In HCT116 cells,

treatment with **PA** at various concentrations for 24 h did not alter hnRNPC mRNA levels. **d** At various time points after **PA** treatment, hnRNPC mRNA levels remained unchanged in HCT116 cells.**e** CETSA assessing the effect of **PA** on the thermal stability of hnRNPC. **f** CETSA evaluating the effect of different concentrations of **PA** on hnRNPC thermal stability at 55 °C. **g** DARTS assay evaluating the effect of **PA** on the protease susceptibility of hnRNPC. **h** DARTS assay assessing the effect of different concentrations of **PA** on hnRNPC protease susceptibility following digestion with 1% Pronase E. **i** hnRNPC overexpression partially reversed the inhibitory effect of **PA** on HCT116 cell proliferation. **j** Overexpression of hnRNPC partially reversed the inhibitory effect of **PA** on HCT116 cell viability. **k** Overexpression of hnRNPC partially reversed the inhibitory effect of PA on the colony-forming ability of HCT116 cells.

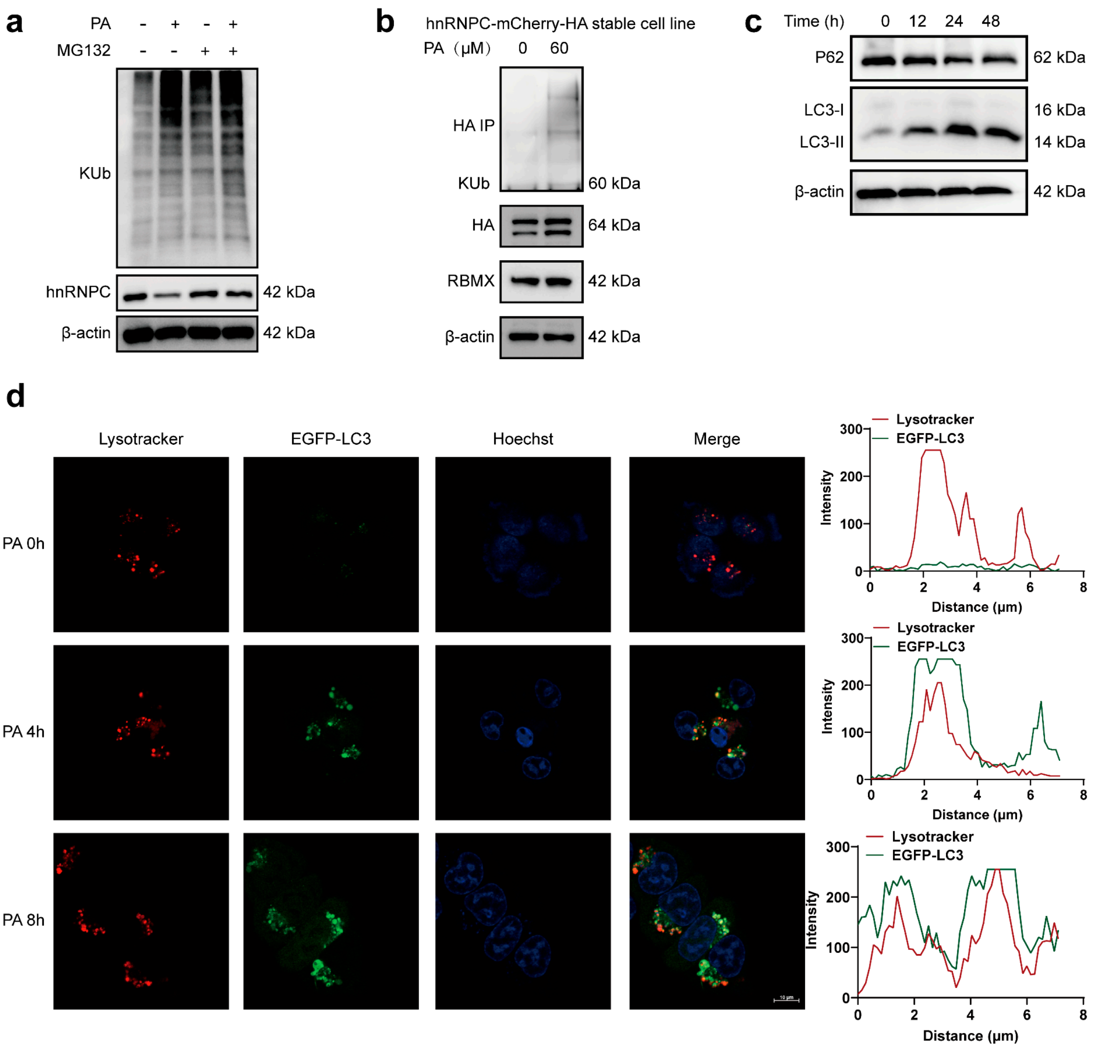

Supplementary Figure S4. In HCT116 cells, **PA** treatment enhances autophagic flux. **a** In HCT116 cells, sequential treatment with **PA** followed by MG132 increased cellular

ubiquitination levels and decreased hnRNPC protein abundance. **b** In the hnRNPC-mCherry-HA stable cell line, treatment with **PA** and MG132 increased hnRNPC ubiquitination levels and RBMX protein abundance. **c** Time-dependent changes in autophagy marker expression in **PA**-treated HCT116 cells. **d** Time-dependent LC3 puncta formation and lysosomal localization in EGFP-LC3-expressing HCT116 stable cells treated with **PA**.
